# Direct Probing of Vibrational Interactions in UiO-66 Polycrystalline Membranes with Femtosecond Two-Dimensional Infrared Spectroscopy

**DOI:** 10.1021/acs.jpclett.2c02509

**Published:** 2022-10-13

**Authors:** Alexander A. Korotkevich, Oleksandr O. Sofronov, Olivier Lugier, Sanghamitra Sengupta, Stefania Tanase, Huib J. Bakker

**Affiliations:** † AMOLF, Ultrafast Spectroscopy, Science Park 104, 1098 XGAmsterdam, The Netherlands; ‡ Functional Materials Group, Van’t Hoff Institute for Molecular Sciences (HIMS), Universiteit van Amsterdam, Science Park 904, 1098 XHAmsterdam, The Netherlands

## Abstract

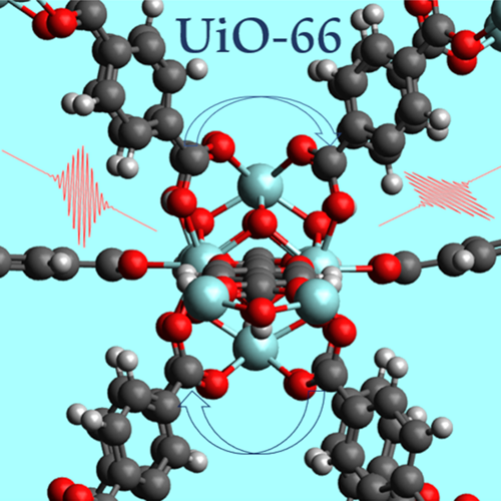

UiO-66 is a benchmark metal–organic framework that holds great promise for the design of new functional materials. In this work, we perform two-dimensional infrared measurements on polycrystalline membranes of UiO-66 grown on c-sapphire substrates. We study the symmetric and antisymmetric stretch vibrations of the carboxylate groups of the terephthalate linker ions and find that these vibrations show a rapid energy exchange and a collective vibrational relaxation with a time constant of 1.3 ps. We also find that the symmetric vibration of the carboxylate group is strongly coupled to a vibration of the aromatic ring of the terephthalate ion. We observe that the antisymmetric carboxylate vibrations of different terephthalate linkers show rapid resonant (Förster) energy transfer with a time constant of ∼1 ps.

Metal–organic frameworks (MOFs) make up a class of crystalline materials in which metal ions or clusters of metal ions are connected by organic linkers to form extended three-dimensional structures. These materials possess well-defined nanopores and nanochannels, and their size and integrity can be tuned over a wide range by varying the metal cations and linkers.^1−4^ As such, different MOF-based functional materials have been prepared and successfully applied in catalysis,^5^ chemical sensing,^6^ separation techniques,^7^ and electrochemistry.^8,9^ An important MOF family is UiO, the members of which consist of Zr_6_O_4_(OH)_4_ building blocks forming a crystal structure by 12-fold coordination with aromatic dicarboxylate linkers.^10,11^ These structures show exceptional stability, even under aggressive conditions, thus making UiOs promising for multiple applications.^12,13^ For example, UiO crystalline membranes have been prepared and used for water desalination, gas separation, storage, and pervaporation.^14−16^ The range of applications can be further extended by preparing UiO type MOFs for which aromatic dicarboxylate linkers are functionalized.^14,17−19^ Such functionalization may thus enable the preparation of stable MOF membranes showing pronounced proton conductivity and/or redox activity, which would be highly promising for designing new fuel cells and electrocatalysis platforms.^18,20^

A crucial parameter determining the properties of UiO membranes is the number density of missing linkers, forming defects in the crystal structure. The deviation from perfect stoichiometry depends on the preparation procedure and often can be controlled, enabling a tuning of the extent of linker–metal interactions.^21−23^ The defect content influences the adsorption and separation properties,^24,25^ the catalytic activity,^26^ and the Brønsted and Lewis acidity^27^ of a UiO membrane. The metal–linker interactions are thus very important for the properties of UiO MOFs, and a detailed understanding of these interactions is a prerequisite for the rational design of new UiO-based materials. Various experimental and theoretical approaches have been used to study the dynamics of UiO derivatives. Recent studies addressed the proton conductivity of UiO MOFs,^28^ and the role of defects in this process,^29^ the dynamics of water adsorbed in the MOF pores,^30^ and the relaxation kinetics after excitation of electronic transitions of the linker.^31,32^

A powerful tool for obtaining structural and dynamical information for chemical systems is two-dimensional infrared spectroscopy (2D-IR). By exciting vibrational modes of molecules or ions with femtosecond infrared pulses, this method discloses unique information about the relaxation kinetics of vibrationally excited states, which in turn provides information about vibrational couplings, inter- and intramolecular energy transfer, and the reorientation dynamics of small molecular species. These processes often strongly depend on the solvation and coordination of the target functional groups. Over the past decade, 2D-IR has been successfully applied to investigate the structural elasticity of UiO-66 prepared as powder samples^33^ and MIL-53(Al).^34^ In this work, we present a 2D-IR investigation of polycrystalline UiO-66 membranes grown on c-sapphire plates. This MOF is the first member of the UiO series and comprises terephthalate (1,4-benzenedicarboxylate, BDC^2–^) as a linker (Figure 1a).

**Figure 1:**
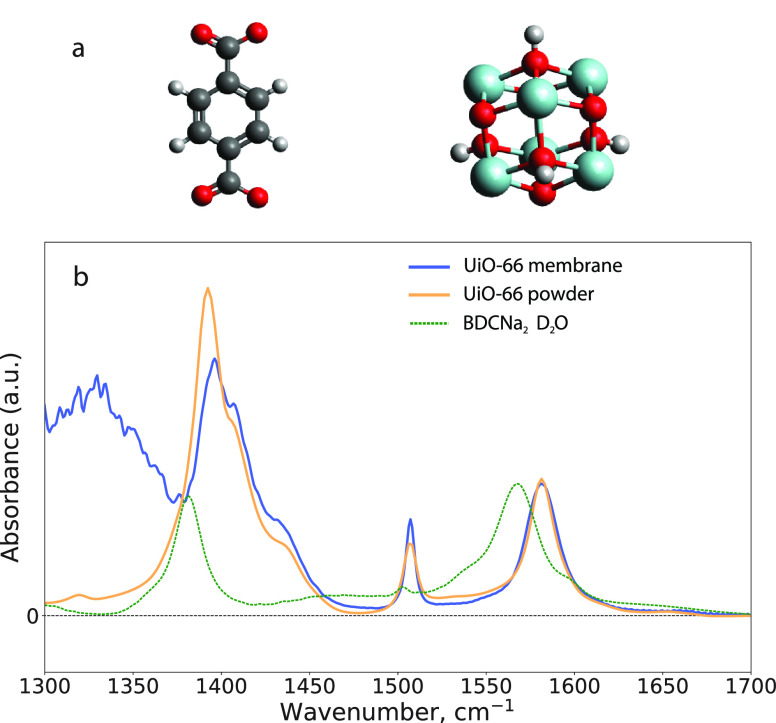
(a) Chemical structures of the terephthalate (BDC^2–^) linker (left) and Zr_6_O_4_(OH)_4_ cluster (right). Black, white, red, and light-blue spheres represent carbon, hydrogen, oxygen, and zirconium atoms, respectively. (b) Linear infrared absorption spectra of UiO-66 membranes grown on a sapphire substrate measured in transmission geometry (blue), UiO-66 powder measured in ATR geometry (orange), and a 0.4 M D_2_O solution of disodium terephthalate (BDCNa_2_, green dashed line). The spectra are normalized with respect to the absorption of the ν_as_ band.

The preparation of UiO-66 membranes on solid substrates is a topic of great current interest. Recently, the preparation of UiO-66 membranes and its derivatives on gold substrates,^14^ sapphire rods,^15^ α-Al_2_O_3_ disks,^35^ and ZrO_2_@γ-Al_2_O_3_ fibers^36^ has been reported. However, to the best of our knowledge, no protocols using flat c-sapphire substrates have been developed. We prepared UiO-66 samples by a solvothermal reaction of ZrCl_4_ and terephthalic acid (BDCH_2_) dissolved in dimethylformamide, using acetic acid as a growth modulator. The sample profilometry showed that the membranes are ∼600–700 nm thick. The crystallinity of the membranes was confirmed with X-ray diffraction analysis. We find that the grains of the polycrystalline membrane have a preferential (111) orientation and that the membrane preserves the cylindrical symmetry. Thermogravimetric analysis (TGA) shows that this preparation method results in ∼42% of missing linkers, which means that the polycrystalline film can be considered a highly defective material. The details of the sample preparation and characterization can be found in the Supporting Information. We further characterize the prepared sample with Fourier transform infrared (FTIR) spectroscopy. In Figure 1b, we compare the infrared absorption spectrum of the prepared UiO-66 membranes with the spectrum of a powder sample. The spectra look very similar in the frequency region above 1400 cm^–1^, which indicates that the local environment of the linkers is very similar in the film and in the powder. We assign the bands at 1585 and 1395 cm^–1^ to the antisymmetric stretch (ν_as_) and symmetric stretch (ν_s_) vibrations, respectively, of the carboxylate anion groups of the linker. The 1510 cm^–1^ band is assigned to the 19a band of the aromatic ring of the linker (further ν_Ph_). This ring mode transforms with the same irreducible representation as the ν_s_.^37−40^ The difference between the spectra at lower frequencies is due to strong sapphire substrate absorption, which precludes a reliable determination of the film absorption values in this frequency region. In our femtosecond 2D-IR experiment, we excite molecular vibrations (ν_s_, ν_Ph_, and ν_as_) of the terephthalate linker of UiO-66 membranes that absorb in the 6 μm region. We measure the excitation-induced absorption change as a function of the excitation and detection frequencies, the waiting time *T* between the excitation and detection pulses, and their mutual polarization direction. The isotropic transient absorption dynamics provide information about the dynamics and mechanism of the interactions involving the excited vibrations. The depolarization dynamics of the transient absorption changes reveal the time scale of intermolecular energy transfer between the carboxylate groups of the linkers. An intrinsic challenge related to femtosecond IR experiments on solid samples is the strong scattering of the excitation light by grains of the studied polycrystalline material. This scattering can overwhelm the signal of the weaker infrared detection pulse, and thus, often special techniques such as using index matching liquids^34^ or advanced phase cycling schemes^33^ have been used to extract reliable data. The UiO-66 membranes that we study scatter light to a much smaller extent than powder samples, and we can sufficiently suppress the remaining scattering by creating a subcycle delay using a wobbler, thus providing another way of overcoming the scattering problem in femtosecond IR studies of MOFs. Sapphire substrates have already been successfully used to grow UiO-66 membranes,^15,16^ and their broad transparency window and the absence of significant nonlinear effects upon interaction with intense infrared pulses enable 2D-IR experiments in transmission geometry.

In Figure 2, we show the 2D-IR spectra of the membrane sample. Due to the limited transparency window of sapphire, we can measure the 2D-IR spectra only when ω_detection_ > 1500 cm^–1^, which excludes a study of the response at ω_detection_ corresponding to ν_s_. However, because the membrane is superposed on the substrate, we are able to detect the transient absorption signals induced by the excitation of ν_s_ at ω_detection_ corresponding to ν_Ph_ and ν_as_.

**Figure 2:**
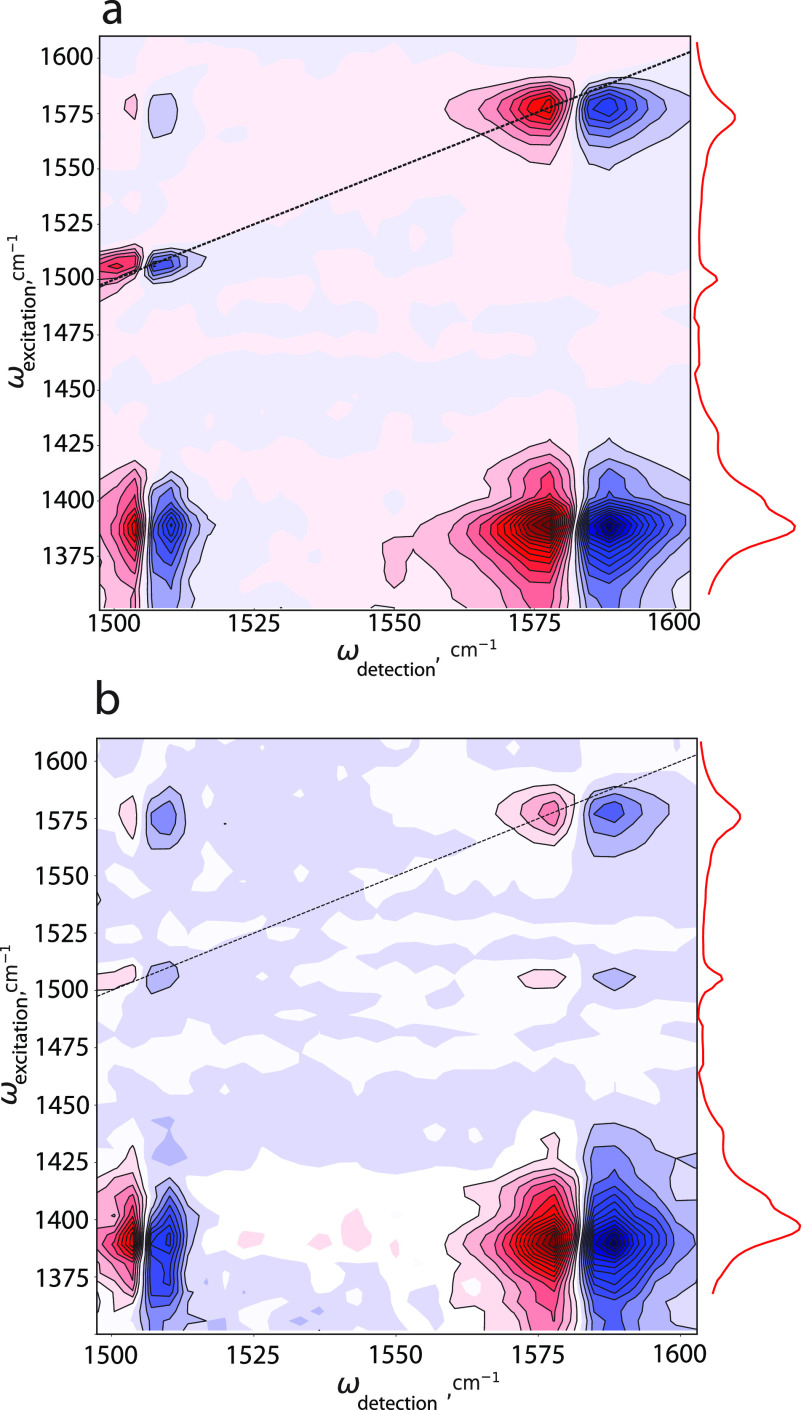
Two-dimensional infrared spectra of UiO-66 membranes grown on sapphire substrates measured at different waiting times. (a) *T* = 0.3 ps, and (b) *T* = 7 ps. The spectra are plotted as a function of the excitation frequency (vertical axis) and the detection frequency (horizontal axis). The inset on the right-hand side shows the linear infrared absorption spectrum to clarify the assignment, and the black dashed diagonal line corresponds to ω_detection_ = ω_excitation_. The spectra are scaled relative to the transient absorption maxima at each waiting time *T*.

In Figure 2a, we observe clear responses on the diagonal line at ω_excitation_ = ω_detection_ = 1510 cm^–1^ and ω_excitation_ = ω_detection_ = 1585 cm^–1^, which correspond to the excitation and detection of ν_Ph_ and ν_as_ vibrations, respectively. We also observe several off-diagonal peaks (cross-peaks) indicating a coupling of the different vibrations. In particular, when ω_excitation_ = 1395 cm^–1^ uphill cross-peaks appear at ω_detection_ = 1510 cm^–1^, indicating vibrational coupling of the ν_s_ mode to the ν_Ph_ mode (ν_s_ → ν_Ph_), and at ω_detection_ = 1585 cm^–1^, indicating vibrational coupling of the ν_s_ mode to the ν_as_ mode (ν_s_ → ν_as_). When ω_excitation_ = 1585 cm^–1^ and ω_detection_ = 1510 cm^–1^, we observe a downhill cross-peak indicating vibrational coupling between ν_as_ and ν_Ph_ (ν_as_ → ν_Ph_).

As one can see in Figure 2b, all signals are still pronounced at a waiting time *T* of 7 ps, but due to vibrational relaxation, their relative strengths have become different. The signal of ν_Ph_ → ν_as_ at ω_excitation_ = 1510 cm^–1^ and ω_detection_ = 1585 cm^–1^ becomes more pronounced than at earlier waiting time. Figures 7 and 8 of the Supporting Information show the amplitude of this signal at earlier waiting times.

To identify the mechanism of vibrational relaxation and the nature of the vibrational coupling in UiO-66 membranes, we measure the dynamics of the diagonal and cross-peak signals. In panels a and b of Figure 3, we show the dynamics of the diagonal peak signal corresponding to ν_as_ and the ν_s_ → ν_as_ uphill cross-peak. We find that both dynamics can be described well with an exponential relaxation with a time constant of ∼1.3 ps to an end level that grows with the same time constant and that shows no further change in amplitude within 200 ps (Figures 8 and 9 of the Supporting Information). The fact that the diagonal ν_as_ and the ν_s_ → ν_as_ cross-peak signals show similar relaxation dynamics points to an ultrafast equilibration between the *v* = 1 states of the ν_as_ and ν_s_ vibrations, which is most likely due to energy transfer occurring within the time scale of the cross-correlate of the excitation and detection pulses. The growth of the end level can be explained by thermal effects. The energy released as a result of the vibrational relaxation affects the shape and position of the vibrational bands, yielding a change in the transient absorption signal that is often termed a hot-state signal. This signal stays constant on the time scale of the time-resolved experiment because its relaxation relies on heat diffusion out of the excited volume, which typically occurs on a microsecond time scale. Next, we take a closer look at the relaxation dynamics of the transient absorption signals involving the ν_Ph_ vibration. In Figure 3c, we show the dynamics of the diagonal peak corresponding to the excitation and detection of ν_Ph_. Compared to the dynamics displayed in panels a and b of Figure 3, this signal decays much more slowly, which indicates that the *v* = 1 state of ν_Ph_ shows a much slower relaxation than the *v* = 1 states of the stretch vibrations of the carboxylate group. The signal decays to a negligible end level, which means that no pronounced thermal effects are observed for the diagonal signal of the ν_Ph_ vibration. In Figure 3d, we present the dynamics of the ν_s_ → ν_Ph_ cross-peak signal. This signal shows a decay that cannot be described well with a single-exponential time constant, and a clear non-zero end level. We find that the dynamics shown in panels c and d of Figure 3 can be consistently described with a model in which the *v* = 1 state of ν_Ph_ has an intrinsic vibrational relaxation time constant of ∼6.7 ps, and in which the ν_Ph_ vibration is coupled to the ν_s_ vibration by two different mechanisms: anharmonic coupling and energy transfer. The anharmonic coupling leads to an instantaneous perturbation of the vibrational potential of ν_Ph_ following the excitation of the ν_s_ vibration. This perturbation leads to a frequency shift of the ν_Ph_ vibration, and thus to a transient absorption change at the detection frequency of the ν_Ph_ vibration that will follow the dynamics of the excitation of the ν_s_ vibration. Therefore, the ν_s_ → ν_Ph_ cross-peak signal will show the 1.3 ps decay time constant of the excited ν_s_ vibration. This component accounts for the fast relaxation component of the cross-peak signal (Figure 3d, blue dashed line). The energy transfer from ν_s_ to ν_Ph_ leads to a contribution to the cross-peak signal that shows a delayed growth with a time constant ∼6.9 ps, followed by a relaxation with a time constant of ∼6.7 ps of the vibrational relaxation of the *v* = 1 state of the ν_Ph_ vibration (Figure 3d, green dashed line). The uphill energy transfer time constant of ∼6.9 ps of ν_s_ to ν_Ph_ is related to a downhill energy transfer time constant of ν_Ph_ to ν_s_ in accordance with the Boltzmann ratio *k*_up_ = *k*_down_ exp(−ℏ*Δω*/*kT*), where *Δω* is the frequency difference between the centers of the absorption bands of ν_s_ and ν_Ph_, which is ∼100 cm^–1^. This energy exchange process is accounted for in describing the diagonal ν_Ph_ signal dynamics in Figure 3c. Finally, the growth of the end level of the cross-peak (Figure 3d, red dashed line) is due to the relaxation of the ν_s_ vibration and the ν_Ph_ vibration, the latter becoming populated as a result of the energy transfer. Given the significantly smaller contribution of the energy transfer to the cross-peak signal, the growth of the end level of the ν_s_ → ν_Ph_ cross-peak signal is dominated by the relaxation of the excited ν_s_ vibration. The signals of the ν_as_ → ν_Ph_ and ν_Ph_ → ν_as_ cross-peaks are much weaker than that of the ν_s_ → ν_Ph_ cross-peak, as one can see from Figure 2a. By further analyzing the dynamics of these cross-peak signals (see Figure 9 of the Supporting Information), we conclude that the vibrational coupling between ν_as_ and ν_Ph_ is much weaker than that between ν_s_ and ν_Ph_. The main coupling mechanism between these vibrations is anharmonic coupling, which implies that the dynamics of the cross-peak signals is determined by that of the excited vibration.

**Figure 3:**
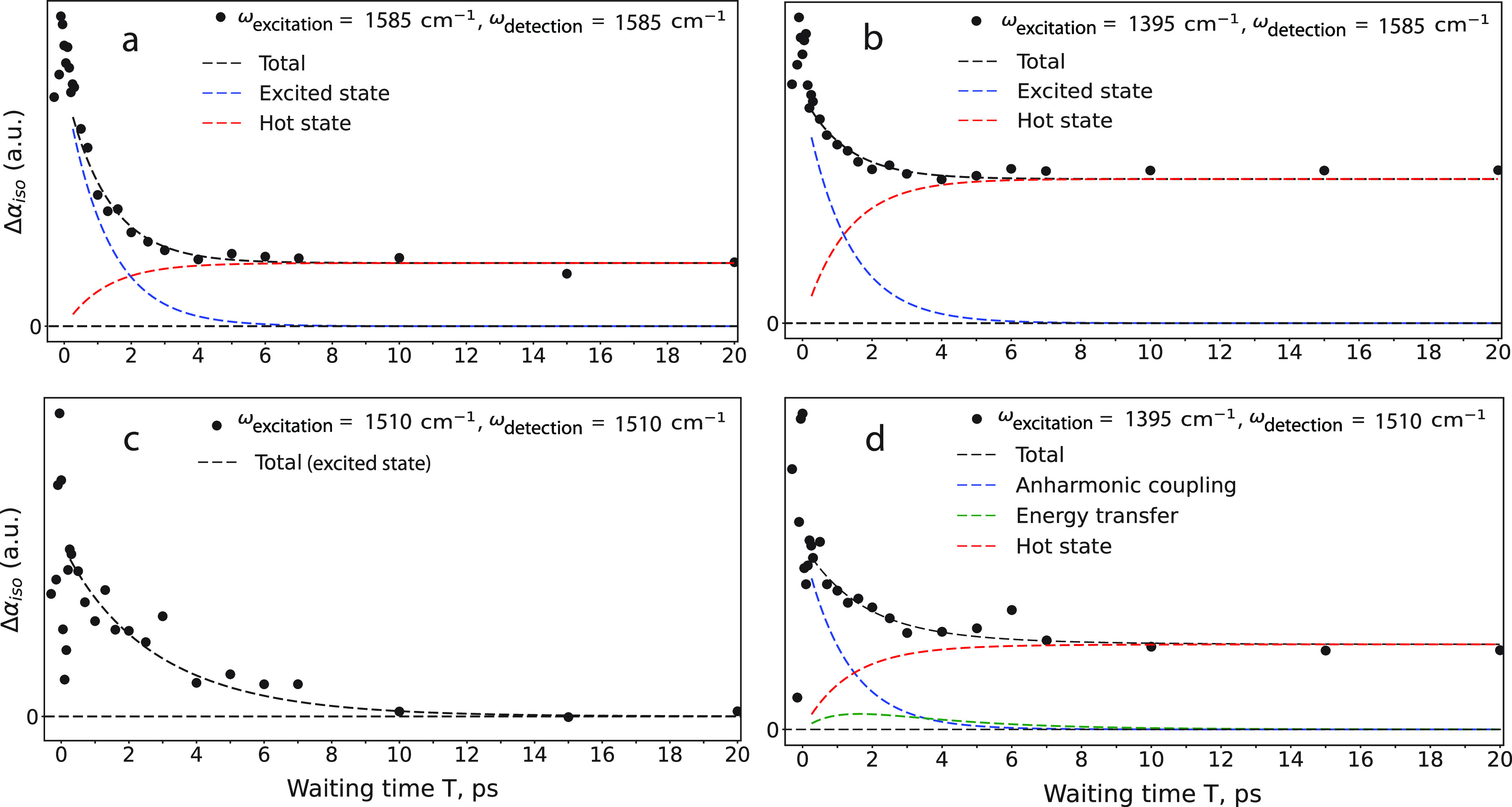
Isotropic transient absorption signals measured for UiO-66 membranes as a function of waiting time *T*, obtained by integrating the 2D signals over an excitation frequency interval of 20–50 cm^–1^ around the maximum frequency of the absorption bands. (a) Exciting ν_as_ and detecting at the maximum frequency of the ν_as_ band (diagonal ν_as_ signal). (b) Exciting ν_s_ and detecting at the maximum frequency of the ν_as_ band (ν_s_ → ν_as_ cross-peak signal). (c) Exciting ν_Ph_ and detecting at the maximum frequency of the ν_Ph_ band (diagonal ν_Ph_ signal). (d) Exciting ν_s_ and detecting at the maximum frequency of the ν_Ph_ band (ν_s_ → ν_Ph_). The black dashed lines represent fits to the dynamics, which contain two or three signal contributions (blue, red, and green dashed lines), according to the model described in the text.

In Figure 4, we show the anisotropy of the excited state of the ν_as_ vibration. To obtain purely the anisotropy dynamics of this excited state, we subtracted the hot-state contributions to the measured *Δα*_∥_ and *Δα*_⊥_ signals prior to constructing the anisotropy parameter. The initial anisotropy value is ∼0.4, which is the value expected for isotropic materials. The anisotropy is observed to decay on a picosecond time scale. Considering that in crystalline material the terephthalate linkers are strongly bound to metal ions, reorientation is not expected to take place on a picosecond time scale. Therefore, we conclude that the observed anisotropy decay is due to excitation transfer between closely spaced and differently oriented linker species. Similarly to Nishida et al.,^33^ we find that these dynamics can be described well by *A*. This expression originates from the Förster model for resonant energy exchange in isotropic materials. We obtain an excitation energy transfer time τ of ∼1 ps.

**Figure 4:**
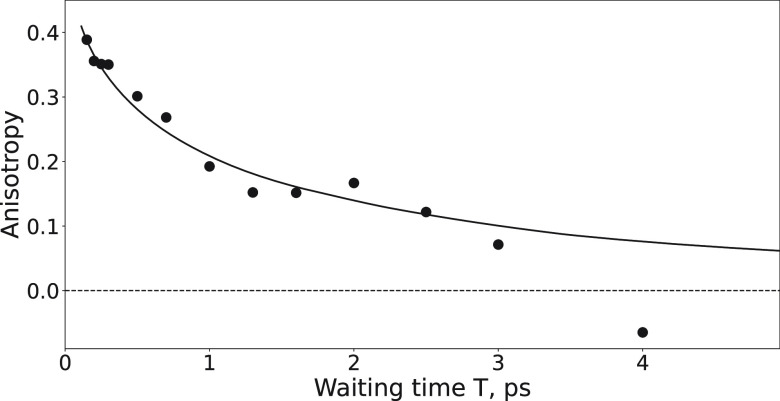
Hot-state corrected anisotropy dynamics of the ν_as_ diagonal peak signal. The solid line represents a fit of the data to a resonant (Förster) energy exchange model.

The infrared absorption spectrum of the terephthalate linker in the UiO-66 structure is quite different from that of the terephthalate dianion in an aqueous solution. First, the cross section of the ν_s_ vibrational mode is almost twice as large as that of the ν_as_ mode in the MOF structure, while in the solution spectrum, these bands have nearly the same amplitude. Interestingly, for disodium terephthalate powder, the cross section of the ν_s_ band is also larger than that of the ν_as_ band, as shown in Figure 4 of the Supporting Information. These findings show that the direct interaction of terephthalate with metal cations leads to a significant redistribution of the charge density and bond strengths in the terephthalate structure, thus significantly altering the transition dipole moments of the carboxylate stretch vibrations. We also find that the absorption band of the ν_Ph_ vibration becomes much stronger in the MOF structure than in terephthalate in aqueous solution. A similar effect is observed for solid disodium terephthalate. The ν_Ph_ vibration has the same symmetry and orientation of its transition dipole as the ν_s_ vibration, and the observed enhancement of the ν_Ph_ vibration is likely due to the interaction between the two vibrations and the increased cross section of the ν_s_ vibration.

We find that the ν_s_ and ν_as_ vibrations of the terephthalate ion show an ultrafast energy equilibration when terephthalate constitutes the linker ion of UiO-66. This result differs from what was observed for the aqueous terephthalate dianion solution. The observations for an aqueous solution indicated the presence of both anharmonic coupling and energy transfer that could be distinguished from each other.^41^ This means that most likely the interaction of the terephthalate linker with the Zr^4+^ cations not only changes the cross sections of the ν_s_ and ν_as_ vibrations but also induces faster energy exchange between these vibrations. The intrinsic vibrational relaxation time constant of ∼1.3 ps that we extract for the equilibrated ν_s_ and ν_as_ vibrations in the solid sample is very close to the vibrational relaxation time constant of these vibrations observed for aqueous terephthalate, suggesting that the collective vibrational relaxation rate of the carboxylate stretch vibrations is only weakly dependent on the ion environment.

Because of the significant enhancement of the cross section of the ν_Ph_ vibration, we also observe clear transient signals associated with this vibrational mode for UiO-66. We also observe pronounced anharmonic coupling and vibrational energy exchange between ν_Ph_ and ν_s_. The interaction between ν_Ph_ and ν_as_ is observed to be much weaker, which can be explained from a simple transition dipole–dipole coupling model.^42^ According to this model, the interaction is stronger for ν_Ph_ and ν_s_ because the transition dipole moments of these vibrational modes are parallel while the transition dipole moments of ν_as_ and ν_Ph_ are perpendicular to each other. The intrinsic vibrational relaxation of the ν_Ph_ mode is observed to be much slower than for the ν_s_ and ν_as_ vibrations, which indicates that this vibration has a quite different relaxation pathway.

In previous time-resolved vibrational spectroscopy studies of MOFs, the relaxation of other types of vibrations was investigated. For loaded MIL-53(Al) MOFs, the stretching vibration of the deuterated bridging hydroxyl termed μ_2_-OD was found to show a relaxation time constant of ∼30–150 ps, depending on the degree of loading.^34^ For the C–O stretching vibration of a carbonyl probe-functionalized linker in UiO-66 powder, a bimodal decay was observed, with a 4–5 ps component and a 40–60 ps component.^33^ The O–D stretching vibration of μ_2_-OD in MIL-53(Al) thus shows a significantly slower relaxation compared to that in aqueous systems.^43−45^ At the same time, the vibrations of carboxylate and carbonyl groups of the linker ions in the MOF structure show relaxation rates that are similar to those observed for these vibrations in aqueous or nonpolar solutions.

We observe that the vibrational relaxation of the carboxylate stretch vibrations leads to a significant residual signal associated with the creation of a long-living hot state. The likely reason for this observation is that the linker density is quite high, which implies that a large amount of thermal energy will accumulate in a limited volume following vibrational excitation and relaxation. Such a heating effect is not observed for an aqueous terephthalate solution and is also much less pronounced in a MIL-53(Al) sample, which can be explained well by the much lower density of μ_2_-OD groups compared to that of terephthalate linkers in the UiO-66 membranes.

The relative contribution of the hot state to the overall signal is quite different for the diagonal and cross-peak signals shown in Figure 3. For the ν_as_ diagonal peak, we obtain a ratio between the decaying and growing contributions of ∼4, while for the ν_s_ → ν_as_ signal, this ratio is ∼1.75. The diagonal signal of ν_Ph_ shows practically no end level, while the cross-peak signal ν_s_ → ν_Ph_ shows a clear non-zero end level. The relative amplitude of the hot-state signal depends on the cross sections of the excited and detected vibrations, as well as on the strength of coupling between them. For the cross-peak signal ν_s_ → ν_Ph_, the end level is relatively high because the initial signal relies on the anharmonic coupling of the two vibrations, which yields a signal weaker than a diagonal signal, while the final signal (the hot-state end level) is determined by the total energy absorbed from the excitation pulse (see the Supporting Information). The excited ν_s_ vibration has a large cross section meaning that a large amount of energy is absorbed, thus leading to a relatively strong thermal effect.

The observation of the hot-state signal means that thermalization of the energy of the excited vibrations results in an immediate red-shift of the vibrational bands of the carboxylate group. Recently, similar red-shifts have been shown to indicate a loosening of metal carboxylate linkages in a broad range of carboxylate-based MOFs.^46^ Hence, our results show that any heat dissipated in the MOF will lead to a loosening of the MOF linkages on a picosecond time scale. This information is important for further mechanistic studies involving MOFs with a broad range of (photo)catalytic functionalities.

We find that the anisotropy of the excitation of the ν_as_ vibration rapidly decays due to resonant energy transfer between differently oriented terephthalate ions. Interestingly, the ν_as_ vibrations of terephthalate ions in aqueous media show quite slow anisotropy dynamics, which can be explained by the much larger mutual distance of the terephthalate ions in solution. In this case, the anisotropy dynamics primarily result from the reorientation of the ion.^41^ It should be noted that the energy transfer between ν_as_ and ν_s_ does not contribute to the anisotropy decay of ν_as_, as the accepting mode (ν_s_) absorbs at a frequency different from that of the probed mode (ν_as_). Hence, this energy transfer leads to a change only in the total transient absorption signal at the ν_as_ frequency, but not of the anisotropic character of this signal.

For MIL-53(Al), a limited anisotropy decay for the μ_2_-OD vibration was observed, which was explained with a wobbling in a cone model, i.e., orientational diffusion within a limited solid angle.^34^ Resonant (Förster) energy transfer has also been observed for the C–O stretching vibration of the carbonyl group that is attached to the aromatic ring of a fraction of terephthalate linkers in UiO-66 powders.^33^ In this study, an acceleration of the anisotropy and central line slope dynamics was observed with an increase in the fraction of functionalized linkers.^33^ The time scale of the observed decay was significantly longer (10–50 ps), even for the highest functionalized linker loadings (14%), than the ∼1 ps transfer time constant that we find for the anisotropy decay of the ν_as_ vibration of the carboxylate group of the terephthalate linker. This difference can be explained well by the fact that the vibrations of the carboxylate groups of the terephthalate linkers are at a much shorter relative distance than the carbonyl groups of a fraction of functionalized terephthalate linkers. In the UiO-66 structure, the nearest neighbor carboxylate groups are attached to the same Zr^4+^ cation, and thus very close to each other. The rate of transfer between the vibrations of the carboxylate groups is thus expected to be quite sensitive to the linker defect content, which will be the subject of future studies.

In summary, in this work we present a new protocol for the preparation of polycrystalline films of UiO-66 on flat c-sapphire substrates. These MOFs consist of Zr^4+^ ions connected by terephthalate linkers. The advantage of this protocol is that the samples are prepared in a single-step solvothermal process that does not involve preparation of the precursors such as Zr-based clusters. Additionally, to the best of our knowledge, no protocols using flat c-sapphire substrates have been reported. The sapphire substrates have a broad transparency window in the optical frequency range that is promising for applications in photocatalysis and electrochemistry. Furthermore, the membranes prepared according to the protocol contain a high concentration of missing linkers. Highly defective materials have been proven to be applicable in absorptive removal of pollutants,^25^ gas separation,^35^ and catalysis.^22^

We studied the prepared UiO-66 films with FTIR spectroscopy and 2D-IR spectroscopy in the fingerprint region. Compared to terephthalate ions in aqueous solution, we find that the cross sections of the ν_s_ carboxylate stretch vibration and ν_Ph_ are strongly enhanced, probably as a result of the interaction with the Zr^4+^ ions. The 2D-IR studies revealed strong vibrational coupling between the ν_s_ and ν_as_ stretch vibrations of the carboxylate group, as well as between the ν_s_ vibration and the ν_Ph_ vibrational modes. The ν_as_ and ν_Ph_ modes show a significantly weaker interaction. We find that the ν_s_ and ν_as_ vibrations show an ultrafast energy exchange and thus relax together with the same effective vibrational relaxation time constant of ∼1.3 ps. The absorption of the ν_Ph_ band shows a slower intrinsic vibrational relaxation with a time constant of ∼6.7 ps. The ν_s_ and ν_Ph_ modes are observed to be anharmonically coupled and to show energy exchange, with an uphill energy transfer time constant of ∼6.9 ps. The measured transient absorption signals show a significant non-zero signal at long delay times that we attribute to a hot state resulting from vibrational relaxation. We find that the anisotropy dynamics of the excitation of the ν_as_ vibrations can be modeled well with a Förster energy exchange model with a time constant of 1 ps. This relatively fast energy transfer can be explained by the proximity of the carboxylate groups of differently oriented terephthalate linkers in the UiO-66 MOF.

## Supporting Information Available

Sample preparation procedure, description and results of characterization of the samples with powder X-ray diffraction, profilometry, and thermogravimetric analysis, description of linear and two-dimensional infrared spectroscopic experiments, additional FTIR spectra 2D-IR spectra and pump-integrated slices, and analysis of the ν_as_ → ν_Ph_ and ν_Ph_ → ν_as_ cross-peak signal dynamics, analysis of the temperature dependence of the FTIR spectra of the UiO-66 membranes and interpretation of signals at long waiting times (PDF)
Transparent Peer Review report available (PDF)
