# Supplementary material for: Direct Probing of Vibrational Interactions in UiO-66 Polycrystalline Membranes with Femtosecond Two-Dimensional Infrared Spectroscopy
Source: J Phys Chem Lett. 2022 Oct 13;13(42):9793–800. doi: 10.1021/acs.jpclett.2c02509 (PMC9620074; doi:10.1021/acs.jpclett.2c02509)
Supplement: Supplementary file 1 — jz2c02509_si_001.pdf [file jz2c02509_si_001.pdf]

Direct Probing of Vibrational Interactions in UiO-66 Polycrystalline Membranes with Femtosecond Two-Dimensional Infrared Spectroscopy. Supporting Information

Alexander A. Korotkevich,*,†,¶ Oleksandr O. Sofronov,†,¶ Olivier Lugier,‡ Sanghamitra Sengupta,† Stefania Tanase,‡ and Huib J. Bakker†

†*Amolf, Ultrafast Spectroscopy, Science Park 104 Amsterdam, Netherlands*

‡*Functional Materials Group, Van't Hoff Institute for Molecular Sciences (HIMS), Universiteit van Amsterdam, Science Park 904, 1098 XH Amsterdam, Netherlands*

¶*The authors contribute equally to this paper.*

E-mail: a.korotkevich@amolf.nl

Sample preparation

We have cut optical grade sapphire wafers (Siegert Wafer, C-plane cut, 0.7 mm thick) with a diamond cutter (AMOLF) in 1×1 cm plates. Zirconium tetrachloride ($ZrCl_4$, Sigma Aldrich anhydrous for synthesis), 1,4-benzenedicarboxylic (terephthalic) acid ($BDCH_2$, Aldrich, 98%), N,N-dimethylformamide (DMF, Sigma-Aldrich, $\geq$ 99.8%) and acetic acid (AcOH, Sigma-Aldrich $\geq$ 99.8%) were used as received. $ZrCl_4$ was constantly stored in a glove-box purged with dry air to avoid hydrolysis of the salt.

We prepared the samples in a single-step anhydrous acidified solvothermal process. Before each synthesis, the plates have been thoroughly wiped with cotton wool wet with acetone and

then sonicated in ethanol for 5 minutes followed by 20 min treatment in a UV-ozone cleaner. After that, the plates were placed in a Teflon sample holder (AMOLF) which was placed in a Teflon beaker of the 23 ml acid digest vessel (Parr instruments). To prepare the reaction mixture, we separately dissolved $ZrCl_4$ and $BDCH_2$ in DMF and sonicated the solutions for 5 minutes. After that, the solutions were combined, AcOH was added and the mixture was again sonicated for 10 minutes. The resulting solution contains the reactants with the following molar ratios: $ZrCl_4$:$BDCH_2$:AcOH:DMF = 1:1:200:400. The solution was after that combined with the clean sapphire plates in the Teflon beaker. We aimed for keeping the sapphire plates on air as short as possible before starting the reaction. After combining the reactants, the reaction vessel was sealed and kept in the oven at 120°C for 24h. Then the vessel was taken out and cooled down in a water bath. The resulting polycrystalline UiO-66 membranes grown on sapphire plates were extracted and purified by consecutive immersion in DMF(1x), water(2x), ethanol (1x) and dried at 50°C. The remaining reaction mixture was transferred to a 15 ml Falcon tube and was further used to extract the UiO-66 powder. The precipitate was isolated by centrifugation of the mixture followed by mechanical supernatant removal. The precipitate was further mixed with 10 ml of fresh DMF for washing the powder and centrifuged again. The process was repeated using consecutively 10 ml of water(2x) and ethanol (1x) for removing the unreacted species and the solvent. Finally, the powder was transferred to a clean vial and dried at 50°C.

X-ray diffraction

To confirm the crystal structure of the prepared membranes, we measured X-ray diffraction patterns with a commercial Bruker D2 Phaser diffractometer using the Cu Kα radiation. While powders measurements were performed using the supplied sample cells, for films grown on substrates a home built sample cell (AMOLF) with adjustable height was used.

Profilometry

We determined the sample thickness with profilometry measurements using KLA Tencor P-7 Stylus Profiler in the AMOLF Nanolab Amsterdam. We made a thin trench on a polycrystalline membrane with a surgical blade and scanned a line of 500 μm having the trench on the way. The trench depth represents the membrane thickness.

TGA

To quantify the defects in the UiO-66 membranes we performed the thermogravimetric analysis (TGA) on powder samples assuming the composition to be similar to the membranes. Before performing the analysis, the powder samples underwent a nitrogen purge in order to remove loosely adsorbed water from the material. The procedure consists of placing the powder for 1 hour in a vial with small holes and an inlet for a nitrogen flow. After that, the powder is rapidly loaded into the TGA apparatus and spends less than 2 min in open atmosphere while weighted. We performed the analysis using a NETZSCH Jupiter STA 449F3 instrument under argon flow (20 mL/min). The samples are heated in aluminium oxide crucible from 35 °C to 700 °C at a rate of 5 K/min. To correct for the effect of buoyancy and other potential artefacts, a reference measurement is done in identical conditions with empty crucibles. Given the TGA curves, we quantified the defect content similarly to the previous UiO-66 study.[1]

Linear infrared spectroscopy

We recorded linear infrared absorption spectra with a commercial Bruker Vertex 80v Fourier-transform infrared spectrometer with a resolution of 2 cm^{-1} in transmission and ATR geometries. For transmission geometry, we used a single bare sapphire substrate for background measurements. For measurements at elevated temperatures, a separate background measurement was performed at each temperature. For ATR measurements, we equipped the

spectrometer with Bruker Platinum ATR accessory and measured background absorption using no sample. The measurements have been performed either at 4.3 hPa or in presence of nitrogen purge to avoid atmospheric absorption.

Two-dimensional infrared spectroscopy

The two-color time-domain 2D-IR setup used in this study has been described in details before.[2,3] In this work, we generate $\sim 250 \quad cm^{-1}$ FWHH pulses centered at ~ 1550 cm^{-1} independently for the detection and the excitation paths. In the excitation path, we use a ZnSe wobbler to suppress the excitation scattering from the sample. After that, we use a Mach-Zehnder interferometer to generate a pulse pair separated by a controlled delay t. The chopper is not used in the 2D-IR experiments since the "excitation-off" configuration is generated in time-domain while scanning the interferometer. The excitation pulse pair is then focused in a sample plane to excite a fraction of vibrations of terephthalate linkers to the first vibrational excited state (v=1). The pump-induced modulation is detected after the controlled delay T with a weaker detection pulse focused in the same spot as the excitation beam. To compensate for pulse-to-pulse energy fluctuations, we use a reference pulse that passes the sample at a slightly different spot and thus does not get perturbed by interacting with the excitation pulse. In the experiments, we used only one side of UiO-66 sample while the second side has been mechanically removed since the light penetration depth does not allow to excite the linkers on the second side.

We thus record the resulting excitation-induced absorption change $\Delta\alpha = \Delta\alpha(T,\ t,\ \omega_{detection})$. Additionally, we simultaneously select probe fraction after the sample polarized either parallel or perpendicular with respect to the excitation polarization and hence $\Delta\alpha_{\parallel}$ and $\Delta\alpha_{\perp}$ are detected. After a Fourier transform with respect to t we get the dependence on the excitation frequency $\Delta\alpha = \Delta\alpha(T,\ \omega_{excitation},\ \omega_{detection})$.

We use the two transient absorption signals to construct the isotropic transient absorption: $\Delta\alpha_{iso} = (\Delta\alpha_{\parallel} + 2\Delta\alpha_{\perp})/3$ the dynamics of which represents depolarization free vi-

brational dynamics. The anisotropy is also constructed using the two transient signals : $R = (\Delta\alpha_{\parallel} - \Delta\alpha_{\perp})/3\Delta\alpha_{iso}$ the dynamics of which represents the time-dependent depolarization.

In Figure 6, we show the absorption spectrum of the sapphire substrates used in this work. As can be seen, the absorption of the substrate starts to increase rapidly for frequencies < 1700 cm^{-1}. At 1500 cm^{-1} the absorbance is around 1, and at 1400 cm^{-1} the absorbance exceeds 2, which means that there is a difference of one order of magnitude in intensity of light that gets transmitted by the substrate. In FTIR measurements, it is still possible to measure absorption around 1400 cm^{-1}, however, as follows from Figure 1, the band corresponding to the symmetric vibration clearly gets distorted as very little intensity of light is transmitted at this frequency and thus a reliable determination of the absorption of this band is not possible. In 2D-IR measurements, we measure the transient change of the absorption induced by excitation. This absorption change is highly sensitive to noise in the linear absorption, and this noise is high because of the limited transmission of the sapphire windows at 1400 cm^{-1}. As a result, the frequency window in which we detect transient absorption changes is limited to frequencies > 1500 cm^{-1}. It should be noted that, although we cannot detect near 1400 cm^{-1}, we can measure 2D-IR spectra involving excitation of the symmetric vibration centered at near 1400 cm^{-1}, because the MOF layer is superimposed on a sapphire substrate and thus the excitation takes place before light is absorbed by the sapphire substrate as described in the main manuscript.

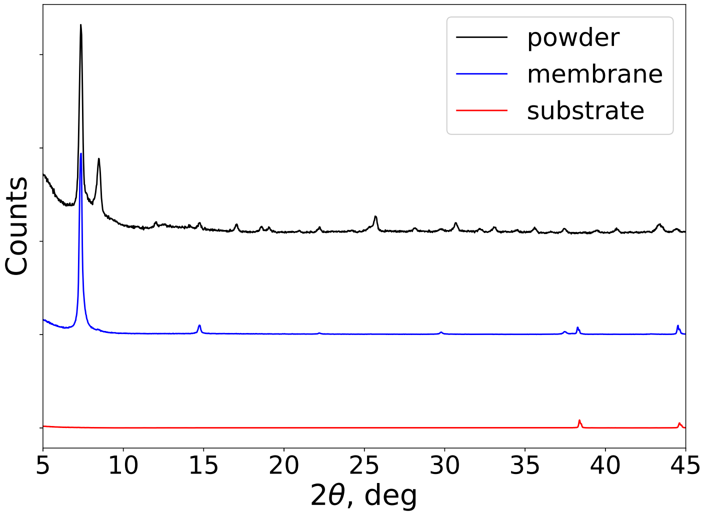

Figure 1: X-ray diffraction patterns of the UiO-66 prepared in the solvothermal process. The absence of the peak at $2\theta = 8.5°$ corresponding to (200)-crystallographic plane of the membrane pattern points at preferential (111)-orientation of the grains of the membrane. With such orientation the cylindrical symmetry of the sample is preserved

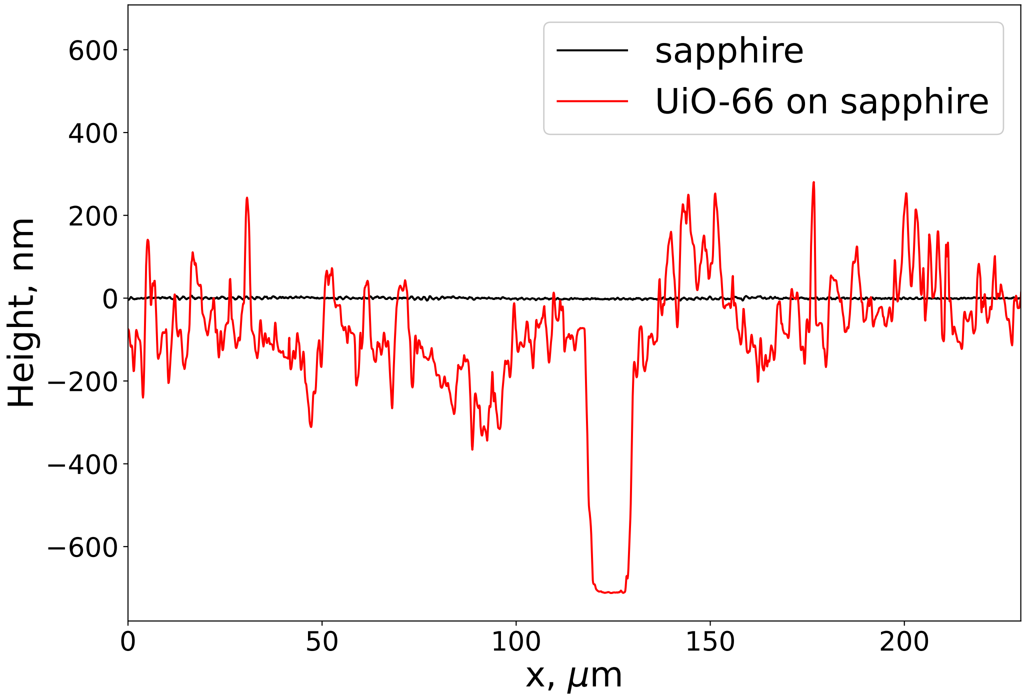

Figure 2: Profile of the UiO-66 polycrystalline membrane. The dip at ∼125 μm corresponds to a trench in a film which shows ∼ 700 nm thickness of the film

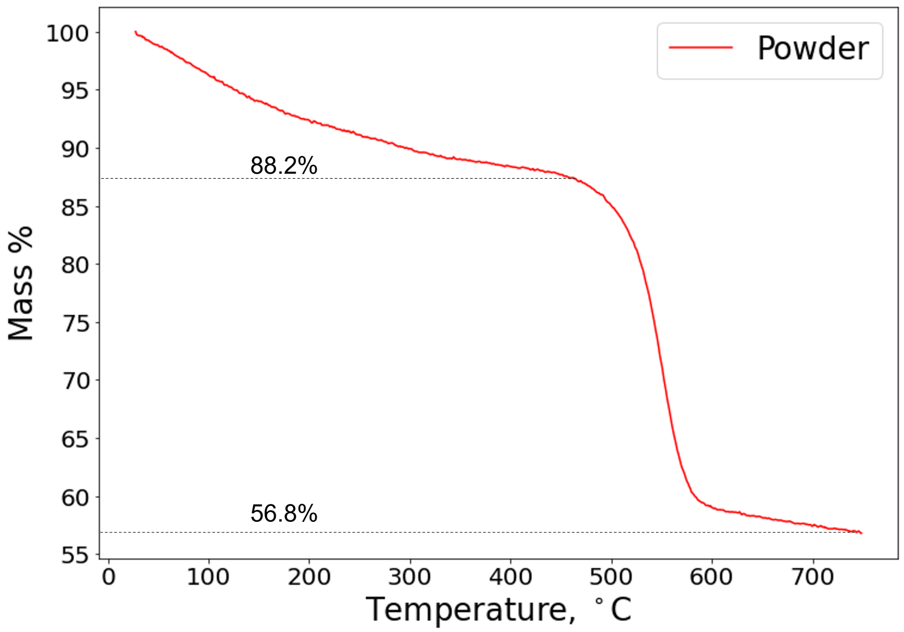

Figure 3: TGA curves for UiO-66 powder. The initial mass-loss of 11.8% corresponds to removal of residual DMF and acetic acid used as a solvent and a crystal growth modulator respectively. After this drop, the MOF is fully desolvated and dehydrated, and the drop of 31.5% at ~ 550 °C corresponds to a total MOF decomposition. For perfect MOF structure, the mass-loss of 54.6% is expected at the last step,[1] which means that the material under study is missing $\sim 42\%$ of the linkers in its structure which is approximately 5 of 12 linkers per metal-oxo cluster

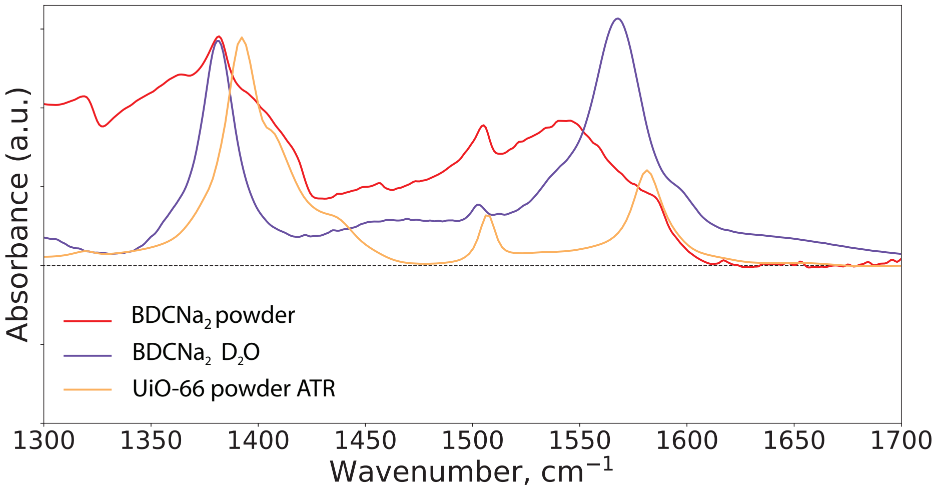

Figure 4: Comparison of infrared absorption spectra of aqueous disodium terephthalate, solid disodium terephthalate and UiO-66

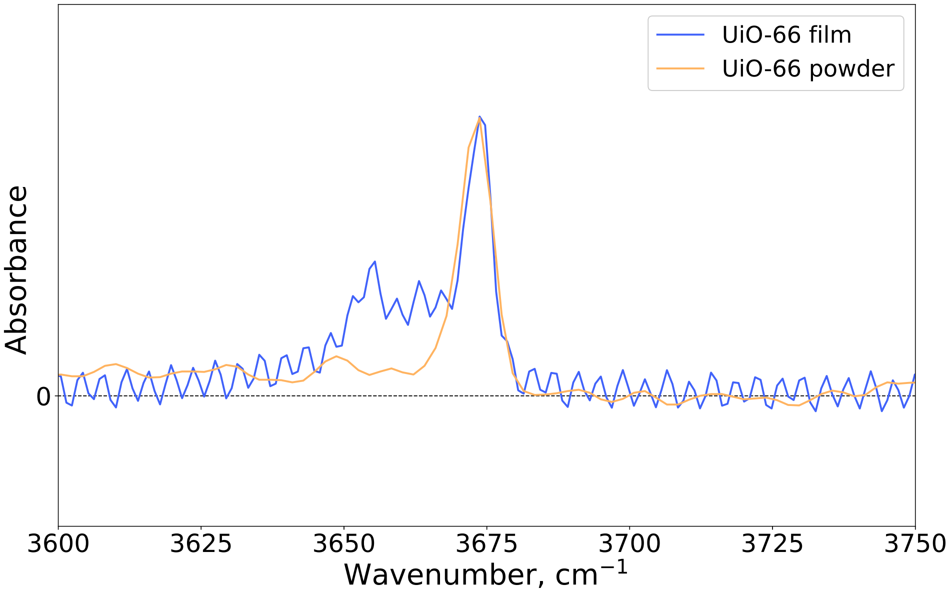

Figure 5: Infrared absorption spectra of a UiO-66 film grown on the sapphire substrates and of the UiO-66 powder. The fringes in the film spectrum are due to interferences in the sapphire window. A peak centered at 3675 cm^{-1} corresponds to the OH groups of the $Zr_6O_4(OH)_4$ clusters, in agreement with previous studies.[1] Interestingly, that another weak band is observed at 3650 cm^{-1}. We assign this band also to OH groups of the metal-oxo clusters. The different frequency can be explained from the fact that the lattice contains defects caused by missing linkers and thus different coordination environment of the OH groups in the clusters.

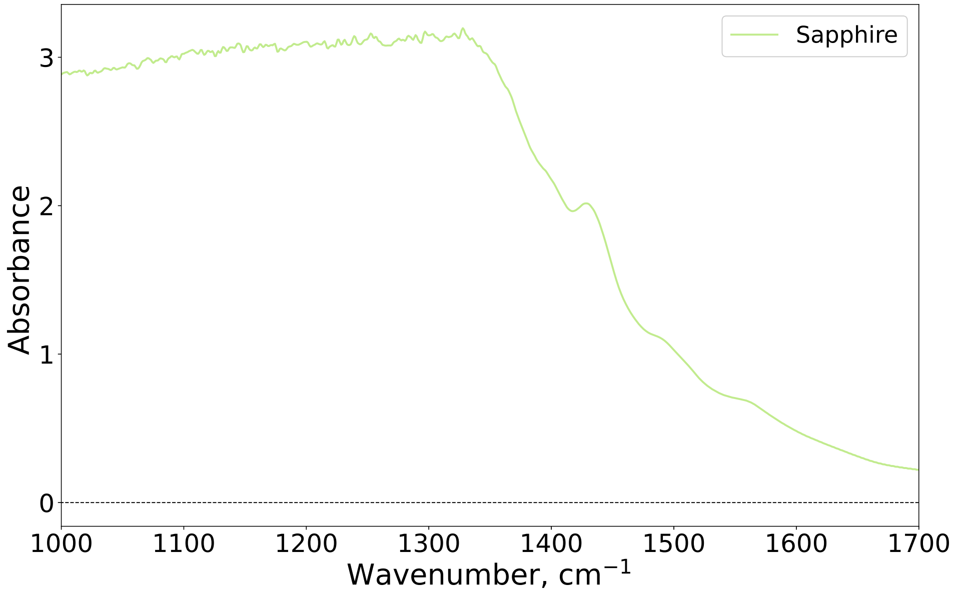

Figure 6: Infrared absorption spectrum of the sapphire substrate.

Analysis of the dynamics of $\nu_{as} \rightarrow \nu_{Ph}$ and the $\nu_{Ph} \rightarrow \nu_{as}$ cross-peak signals

The dynamics of $\nu_{as} \rightarrow \nu_{Ph}$ and $\nu_{Ph} \rightarrow \nu_{as}$ cross-peak signals are analyzed in Figure 9. As can be clearly seen, $\nu_{as} \rightarrow \nu_{Ph}$ and $\nu_{Ph} \rightarrow \nu_{as}$ cross-peaks show almost no relaxation and predominately consist of the weak non-relaxing end-level. These signals appear to be much weaker than $\nu_s \rightarrow \nu_{Ph}$ cross-peak as can be seen from Figure 2 of the main text. Moreover, neither the dynamics of ν_{as} diagonal signal nor that of the $\nu_s \rightarrow \nu_{as}$ cross-peak signal seem to be significantly influenced by interaction with the ν_{Ph} showing similar dynamics. From that we conclude, that the interaction between the ν_{Ph} and the ν_{as} is significantly weaker than that of the ν_{Ph} and the ν_s. We find that dynamics of the $\nu_{Ph} \rightarrow \nu_{as}$ can be described as a result of combination of decaying contribution identical to the dynamics of ν_{Ph} diagonal peak signal followed by hot state contribution growing as fast as the decay. The dynamics of $\nu_{as} \rightarrow \nu_{Ph}$ cross-peak is also described by combining a decaying and a growing contributions but the time constant for this process coincides with that of the relaxation time constant

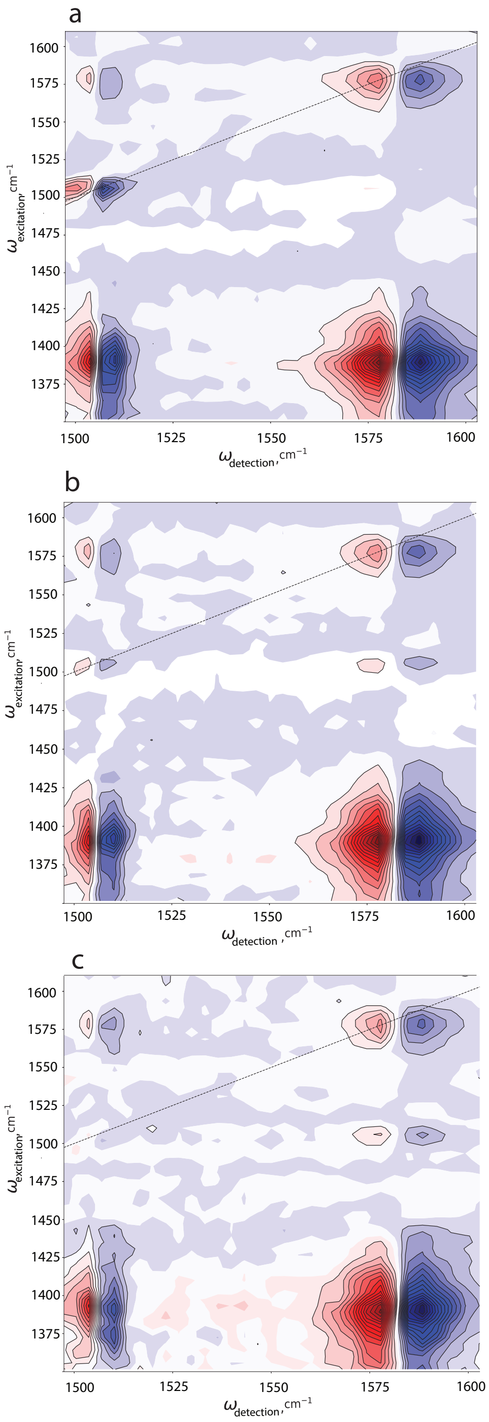

Figure 7: Two-dimensional infrared spectra of UiO-66 membranes grown on sapphire substrates at different waiting times a. T = 2 ps b. T = 10 ps c. T = 200 ps. The spectra are plotted as a function of the excitation frequency (vertical axis) and the detection frequency (horizontal axis). The black dashed diagonal line corresponds to $\omega_{detection} = \omega_{excitation}$. The spectra are scaled relative to the transient absorption maxima at each waiting time T.

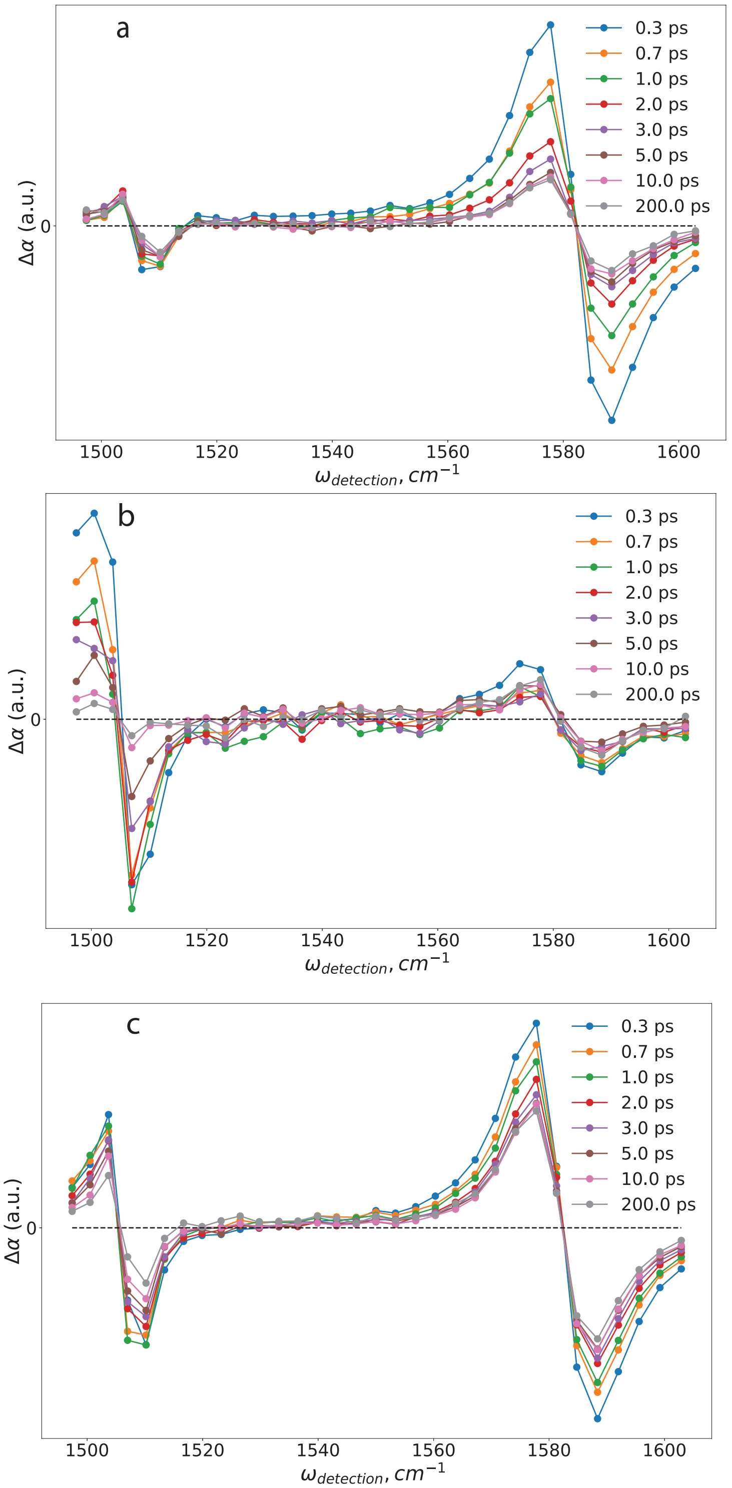

Figure 8: Isotropic transient absorption spectra measured for UiO-66 membranes as a function of waiting time T, and detection frequency $\omega_{detection}$ obtained by integrating the 2D signals over an excitation frequency interval of 20-50 cm^{-1} a. Exciting ν_{as} b. Exciting ν_{Ph} c. Exciting ν_s

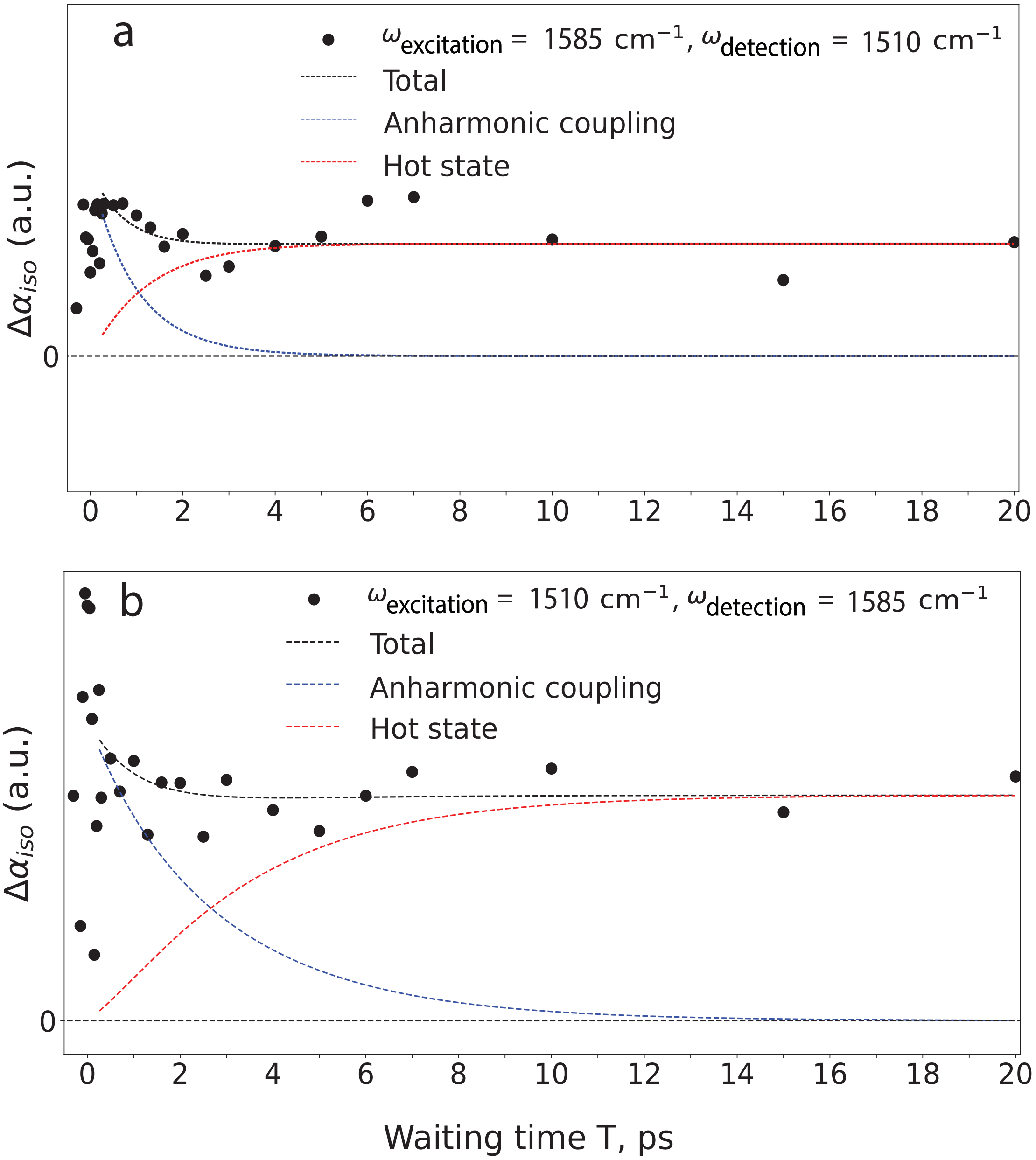

Figure 9: Isotropic transient absorption signals measured for UiO-66 membranes as a function of waiting time T, obtained by integrating the 2D signals over an excitation frequency interval of 20-50 cm^{-1} around the maximum frequency of the absorption bands. Figure a: Exciting ν_{as} and detecting at the maximum frequency of the ν_{Ph} band ($\nu_{as} \rightarrow \nu_{Ph}$ cross-peak signal); b: Exciting ν_{Ph} and detecting at the maximum frequency of the ν_{as} band ($\nu_{Ph} \rightarrow \nu_{as}$ cross-peak signal)

of ν_{as}. The fact that the decaying contribution has the same dynamics as excited vibration points at anharmonic coupling between ν_{Ph} and ν_{as} vibrations.

Analysis of the temperature dependence of the FTIR spectra of the UiO-66 membranes and interpretation of the signals at long waiting times

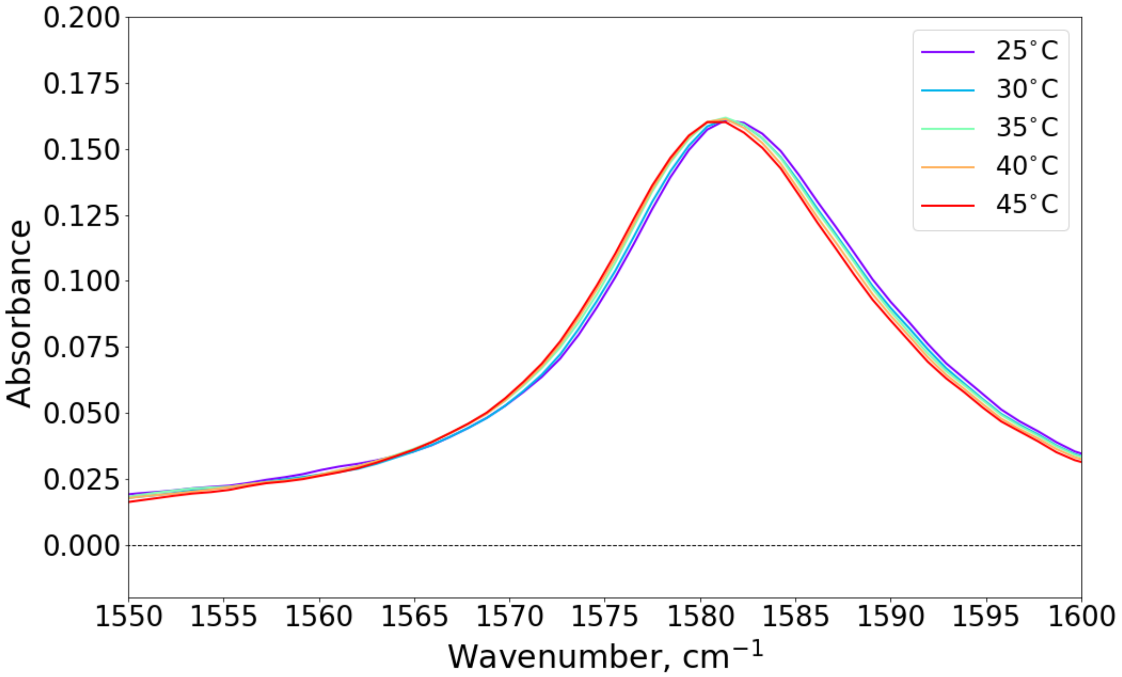

Figure 10: Infrared absorption spectra of UiO-66 membranes at elevated temperatures

To corroborate the thermal nature of the transient signals at long waiting time, we have performed a study of temperature dependence of the membrane FTIR spectrum. In Figure 10 we show the spectra detected at elevated temperatures in the frequency region corresponding to the ν_{as} vibration. As can be clearly seen from Figure 10, increasing the temperature leads to a shift of the maximum of the band to lower frequencies without significantly changing the absorption cross-section. The magnitude of the shift is on the order of a few cm^{-1} and proportional to the temperature increase. By subtracting the spectrum detected at room temperature from the spectra detected at elevated temperature, we obtain the thermal difference spectra that we show in Figure 11. Comparison of Figure 11 with Figure 8 shows

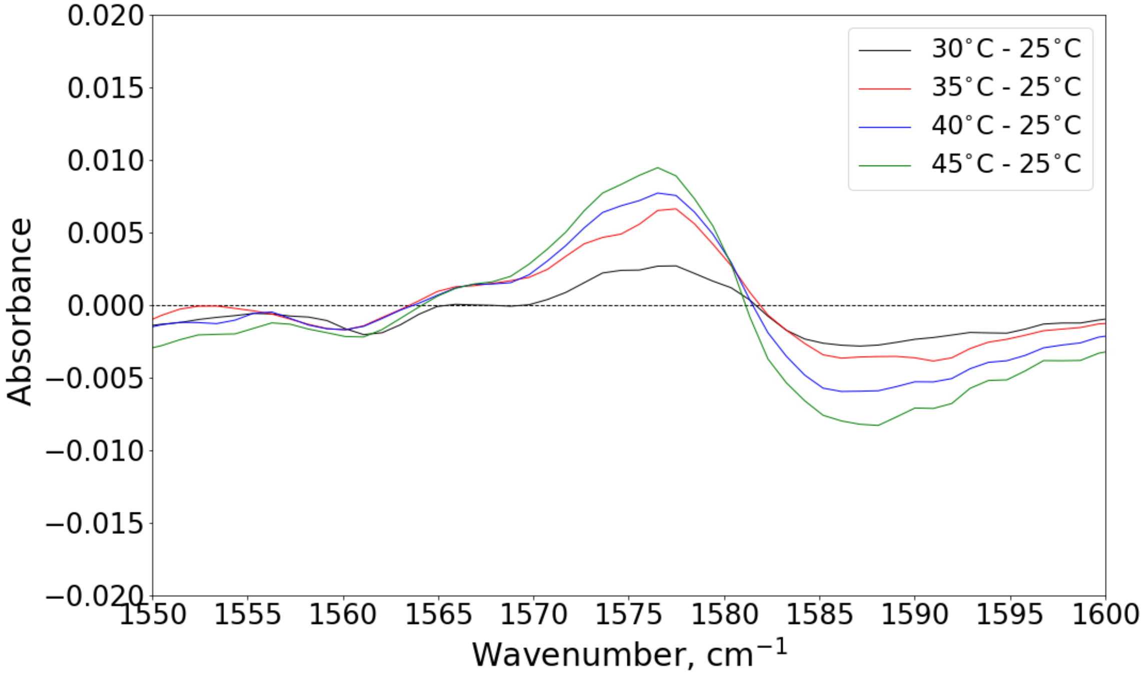

Figure 11: Thermal difference of the infrared absorption spectra of UiO-66 membranes displayed in Figure 10

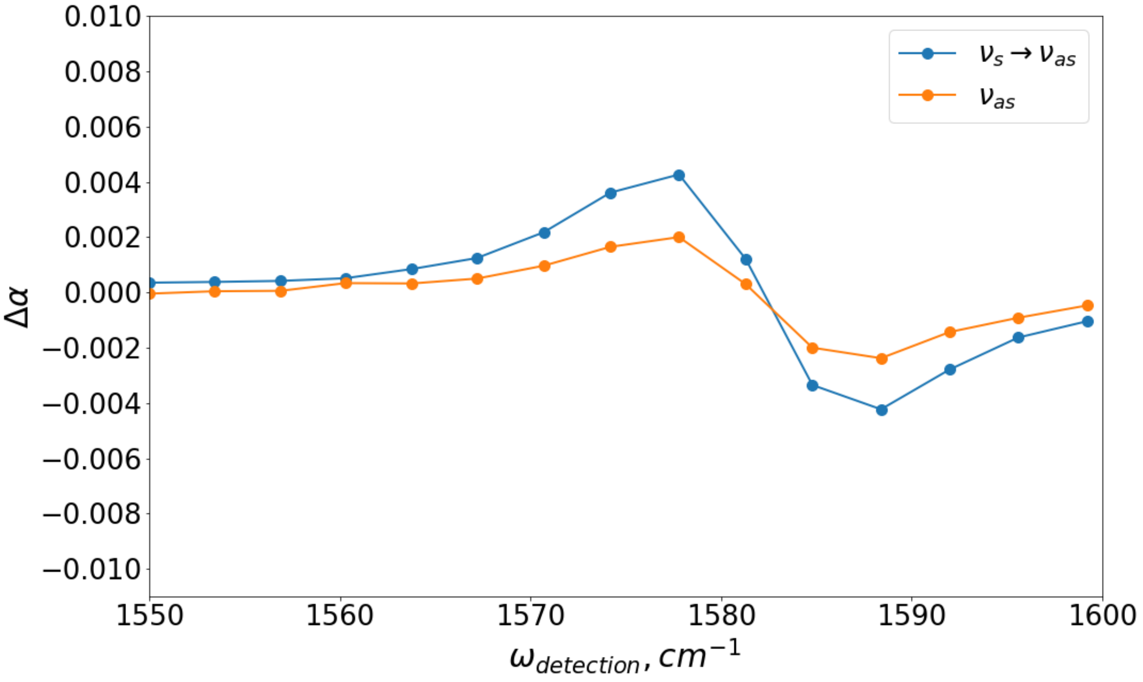

Figure 12: Isotropic transient absorption spectra measured for UiO-66 membranes at waiting time T = 200 ps for $\nu_s \rightarrow \nu_{as}$ cross-peak signal (blue), ν_{as} diagonal signal (orange). Note that decimal logarithm was used to calculate the transient absorption

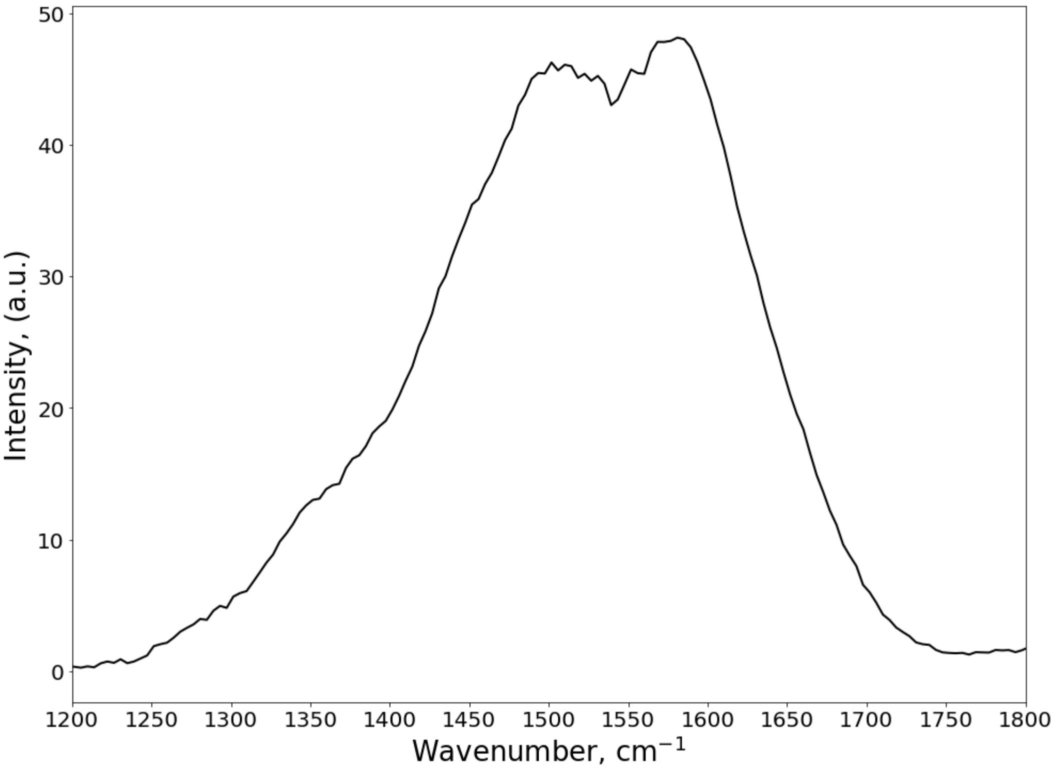

Figure 13: Spectral profile of the excitation pulse

that the shape of the thermal difference spectrum is very similar to the difference absorption spectra observed at waiting times > 10 ps with 2D-IR spectroscopy. As the shift of the band is much smaller than the bandwidth, subtraction of the spectra yields an anti-symmetric dispersive shape of which the amplitude scales with the magnitude of the shift. This shape is similar to that of the excited state spectrum because the diagonal anharmonicity is also much smaller than the width of the absorption band. We calculated the temperature change in the excited volume using the following expression: $\Delta T = E_{absorbed}/(c \cdot m)$, where ΔT is the temperature change, $E_{absorbed}$ is the excitation energy absorbed by the sample, c is the heat capacity and m is the mass of the volume that is excited. The absorbed energy was calculated by integration:

$$E_{absorbed} = \int_{\omega_{min}}^{\omega_{max}} d\omega((1 - 10^{-A(\omega)})S(\omega)) \tag{1}$$

where $A(\omega)$ is the sample absorbance determined from FTIR spectra, $S(\omega)$ the spectrum

of the excitation pulse shown in Figure 13. We used $\omega_{min} = 1350$ cm^{-1}, $\omega_{max} = 1450$ cm^{-1} and $\omega_{min} = 1540$ cm^{-1}, $\omega_{max} = 1610$ cm^{-1} for ν_s and ν_{as} vibrations respectively. The excitation pulse energy of $\sim$ 5 μJ was used for normalization of the integral. To calculate the mass of the excited volume we used the beam diameter at the sample position of $\sim$ 200 μm, the sample thickness of 700 nm (see Figure 2), UiO-66 density of 1200 kg/m^3[4] and a heat capacity of 1000 J/(kg·K).[5] With these parameters, we calculate the increase in temperature ~10K for excitation of the ν_{as} vibration and ~20K in the case of excitation of the ν_s vibration. In Figure S12, we show pump-averaged transient spectra corresponding to the diagonal ν_{as} peak ($\omega_{excitation} = \omega_{detection} = 1585$ cm-1) and the $\nu_s \rightarrow \nu_{as}$ cross-peak ($\omega_{excitation} = 1395$ cm^{-1} , $\omega_{detection} = 1585$ cm^{-1}). Comparing Figures 11 and 12 it is clearly seen that the transient absorption spectra observed at long waiting times shown in Figure 12 match quite well with thermal difference spectra in Figure 11. This result corroborates the assignment of the transient absorption spectra at waiting times >10 ps to a heating effect, i.e. completed vibrational relaxation and thermal equilibration in the UiO-66 film.

References

(1) Valenzano, L.; Civalleri, B.; Chavan, S.; Bordiga, S.; Nilsen, M. H.; Jakobsen, S.; Lillerud, K. P.; Lamberti, C. Disclosing the Complex Structure of UiO-66 Metal- Organic Framework: a Synergic Combination of Experiment and Theory. *Chem. Mater.* **2011**, *23*, 1700–1718.

(2) Selig, O.; Siffels, R.; Rezus, Y. L. A. Ultrasensitive Ultrafast Vibrational Spectroscopy Employing the Near Field of Gold Nanoantennas. *Phys. Rev. Lett.* **2015**, *114*, 233004–1–233004–5.

(3) Selig, O.; Cunha, A. V.; van Eldijk, M. B.; van Hest, J. C. M.; Jansen, T. L. C.; Bakker, H. J.; Rezus, Y. L. A. Temperature-Induced Collapse of Elastin-like Peptides Studied by 2DIR Spectroscopy. *J. Phys. Chem. B* **2018**, *122*, 8243–8254.

(4) Connolly, B.; Aragones-Anglada, M.; Gandara-Loe, J.; Danaf, N.; Lamb, D.; Mehta, J. P.; Vulpe, D.; Wuttke, S.; Silvestro-Albero, J.; Moghadam, P. Z.; Wheatley, A.; Fairen-Jimenez, D. Tuning Porosity in Macroscopic Monolithic Metal Organic Frameworks for Exceptional Natural Gas Storage. *Nat. Commun.* **2019**, *10*, 2345, (1–11).

(5) Wieme, J.; Vandenbrande, S.; Lamaire, A.; Kapil, V.; Vanduyfhuys, L.; Van Speybroeck, V. Thermal Engineering of Metal–Organic Frameworks for Adsorption Applications: A Molecular Simulation Perspective. *ACS Appl. Mater. Interfaces* **2019**, *11*, 38697–38707.
