# Supplementary material for: Direct Probing of Vibrational Interactions in UiO-66 Polycrystalline Membranes with Femtosecond Two-Dimensional Infrared Spectroscopy
Source: J Phys Chem Lett. 2022 Oct 13;13(42):9793–800. doi: 10.1021/acs.jpclett.2c02509 (PMC9620074; doi:10.1021/acs.jpclett.2c02509)
Supplement: Supplementary file 2 — jz2c02509_si_002.pdf [file jz2c02509_si_002.pdf]

jz-2022-02509b.R1

Name: Peer Review Information for "Direct Probing of Vibrational Interactions in UiO-66 Polycrystalline Membranes With Femtosecond Two-Dimensional Infrared Spectroscopy"

First Round of Reviewer Comments

Reviewer: 1

Comments to the Author

In this paper the authors report the results on 2D IR spectroscopy of UiO-66 polycrystalline membranes. They concentrate on three modes: the symmetric and antisymmetric vibrations of the linker and the aromatic ring vibrations. On the basis of the results the authors conclude on the coupling of different vibrations and vibrational relaxations

I started reading the manuscript with high expectations because I had read some interesting papers by some of the coauthors. Unfortunately, by the time I got to the end, I was a bit disappointed.

My general impression is that the paper, while containing some interesting observations, is not of broad interest and is better suited to a more specialized journal (e.g. Vibrational spectroscopy or Spectrochimica Acata A).

In any case, when the manuscript is re-submitted, there are some things that should be taken into account.

At the beginning of the description of the results the authors assign a band at 1385 cm-1 to the symmetric stretching modes of the carboxylate linker. However, the spectra shown in Fig. 1 indicate that the band is located between 1395 and 1400 cm-1. This is also supported by numerous literature data. In fact, the band at 1385 cm-1 in Fig. 1 rather belongs to the spectrum of the Na2(BDC) solution.

Because the band at ca. 1400 cm-1 is well detected, I am confused by the following statement: “Due to the limited transparency window of sapphire, we can only measure 2D-IR spectra with ω detection > 1500 cm-1”. In case of real hindrance, this should be better explained.

Another think is that the authors did not present the spectra in the (OH) stretching region, although it is well known that the hydroxyl coverage of UiO-66 is very important for its properties.

A Table of Content picture is missing.

There is no discussion of how the obtained results can be useful to researchers working with MOFs who are not specialists in spectroscopy.

In the last paragraph of the paper, after discussing the effect of defects on the spectra, the authors suddenly start a text that looks like Conclusions.

Reviewer: 2

Comments to the Author

The article reports the use of FTIR and 2D-IR spectroscopy to study the vibrational dynamics of the UiO-66 MOF, formed as a membrane on a sapphire substrate. The authors report fast vibrational relaxation of carboxylate stretching vibrations to a 'hot state' along with coupling of these modes to a ring vibrational mode of terephthalate groups. Finally, Forster energy transfer between different linkers is identified.

MOF-based systems are of significant interest to the chemical community as a result of a growing range of applications, which the authors outline in the introduction to the article. There has however been comparatively little work done on understanding the inter and intramolecular processes that take place within their complex structures. This means that studies such as this will be of interest to both the spectroscopy and dynamics community as well as a broad range of chemists using MOFs. As such, the work is worthy of publication in JPC Letters.

Prior to publication there are a small number of minor points which the authors might wish to address:

1) To make the paper more accessible to non-experts, a diagram of the relevant molecular structures would be helpful in following the spectroscopic interpretations.

2) The formatting of Figure 2 was not ideal in my version – in particular the left-hand side of the 2D-IR spectra appear to be cut off by the axis labels.

3) The assignment of the features left at long waiting times to a 'hot state' (assumed to mean generic thermal heating following vibrational relaxation) seems reasonable given the observed dynamics. It is however noticeable that the spectra at waiting times of 200 ps and 7 ps are virtually identical to the one recorded at 2 ps, except for some changes in amplitude caused by energy transfer. This suggests that the 'hot state' has an identical spectrum to that of the vibrationally excited material and makes it hard to differentiate the proposed mechanism of relaxation and thermal heating from a scenario in which vibrational relaxation proceeds via fast energy transfer (1.3 ps) and a very slow (> 200ps) vibrational relaxation. How do the authors rule out the second scenario? A study of the FTIR spectrum of the membrane as a function of temperature could be used to show the result of increased T on the carboxylate bands for comparison with the 200 ps data. Ordinarily it would be expected that a carboxylate band would shift to higher frequencies at elevated temperature, which would give a different spectral profile to that of a vibrationally-excited one.

4) Related to 3) it is stated in the discussion that 'the final signal (the hot-state end level) is determined by the total energy absorbed from the excitation pulse.' Has this been experimentally verified?

5) There are several very long passages of text (e.g. p4&5), which would be made easier to read by greater use of paragraphs.

Author's Response to Peer Review Comments:

Dear Professor Editor,

We hereby would like to resubmit our manuscript jz-2022-02509b entitled "*Direct Probing of Vibrational Interactions in UiO-66 Polycrystalline Membranes with Femtosecond Two-Dimensional Infrared Spectroscopy*". We thank you for your prompt handling of our manuscript and the reviewers for their suggestions for improvement. In a revised version of the manuscript, we have addressed the reviewers' concerns and implemented the suggested improvements. We also reformatted the manuscript according to the requested non-scientific changes and journal guidelines. Below you find our reply to the report in which we also explain how we changed the manuscript accordingly.

With kind regards,

Alexander A. Korotkevich, Oleksandr O. Sofronov, Olivier Lugier, Sanghamitra Sengupta, Stefania Tanase and Huib J. Bakker

04.10.2022, Amsterdam

Itemized response

Reviewer 1

Reviewer's question

At the beginning of the description of the results the authors assign a band at 1385 cm-1 to the symmetric stretching modes of the carboxylate linker. However, the spectra shown in Fig. 1 indicate that the band is located between 1395 and 1400 cm-1. This is also supported by numerous literature data. In fact, the band at 1385 cm-1 in Fig. 1 rather belongs to the spectrum of the Na2(BDC) solution.

Authors' response

We thank the reviewer for this remark. The band corresponding to the symmetric vibration of the linker is indeed located between 1395 and 1400 cm^{-1} for UiO-66 powder and UiO-66 membrane, while it is centered at 1385 cm^{-1} for the linker dissolved in D_2O, as shown in Figure 1. Hence, the way we referred to this band in the previous version of the manuscript was a typo that was repeated throughout the text and in Figure 3. In the revised manuscript we corrected this error. We now state that the band of the symmetric vibration of UiO-66 is located between 1395-1400 cm^{-1}.

Actions taken

We corrected the assignment of the symmetric vibration of UiO-66 throughout the text and in the Figure 3.

Reviewer's question

Because the band at ca. 1400 cm-1 is well detected, I am confused by the following statement: "Due to the limited transparency window of sapphire, we can only measure 2D-IR spectra with ω detection > 1500 cm-1". In case of real hindrance, this should be better explained.

Authors' response

We thank the reviewer for this question. In Figure 6 of the Supporting Information, we show the absorption spectrum of the sapphire substrates used in this work. As can be seen, the absorption of the substrate starts to increase rapidly for frequencies < 1700 cm^{-1}. At 1500 cm^{-1} the absorbance is around 1, and at 1400 cm^{-1} the absorbance exceeds 2. In 2D-IR measurements, we measure the transient change of the absorption induced by the excitation. This transient absorption change is highly sensitive to noise in the linear absorption, and this noise is high because of the limited transmission of the sapphire windows at 1400 cm^{-1}. As a result, the frequency window in which we ***detect*** transient absorption changes is limited to frequencies >1500 cm^{-1}. It should be noted that, although we cannot detect near 1400 cm^{-1}, we can measure 2D-IR spectra involving ***excitation*** of the symmetric vibration centered at near 1400 cm^{-1}, because the MOF layer is superposed on the sapphire substrate and thus the excitation takes place before light is absorbed by the sapphire substrate.

Actions taken

We added the following paragraph to the Supporting Information.

"In Figure 6 we show the absorption spectrum of the sapphire substrates used in this work. As can be seen, the absorption of the substrate starts to increase rapidly for frequencies < 1700 cm^{-1}. At 1500 cm^{-1} the absorbance is around 1, and at 1400 cm^{-1} the absorbance exceeds 2, which means that the transmission is less than 1% of the light is transmitted at this frequency by the substrate. As shown in Figure 1, this frequency-dependent absorption leads to a strong distortion of the band corresponding to the symmetric vibration. In 2D-IR measurements, we measure the transient change of the absorption induced by excitation. This absorption change is highly sensitive to noise in the linear absorption, and this noise is high because of the limited transmission of the sapphire windows at 1400 cm^{-1}. As a result, the frequency window in which we detect transient absorption changes is limited to frequencies >1500 cm^{-1}. It should be noted that, although we cannot detect near 1400 cm^{-1}, we can measure 2D-IR spectra involving excitation of the symmetric vibration centered at 1400 cm^{-1}, because the MOF layer is superposed on the sapphire substrate and thus the excitation takes place before light is absorbed by the sapphire substrate."

Reviewer's question

Another think is that the authors did not present the spectra in the (OH) stretching region, although it is well known that the hydroxyl coverage of UiO-66 is very important for its properties.

Authors' response

We did measure spectra in the OH stretching region for the UiO-66 membrane and powder samples. We included these spectra in the Supporting Information of the revised manuscript. In these spectra we observe a peak centered at 3675 cm^{-1} that we assign to the stretch vibrations of OH groups of the $Zr_6O_4(OH)_4$ clusters, in agreement with previous studies (see for instance Chem. Mater. 2011, **23**, 7, 1700–1718). Interestingly, we observe an additional weak band at 3650 cm^{-1}. We assign this band also to the stretch vibrations of OH groups of metal-oxo clusters. The different frequency can be explained from the fact that the lattice contains defects caused by missing linkers, thus yielding a different coordination environment of the OH groups in the clusters.

Actions taken

We added Figure 5 to the Supporting Information that contains spectra in the OH stretching region, and we added the following paragraph to the caption of the Figure discussing these spectra. "Infrared absorption spectra of a UiO-66 film grown on a sapphire substrate and of UiO-66 powder. The fringes in the film spectrum are due to interferences in the sapphire window. The peak centered at 3675 cm^{-1} is assigned to the stretch vibrations of OH groups of the $Zr_6O_4(OH)_4$ clusters, in agreement with previous studies (Chem. Mater. 2011, 23, 7, 1700–1718). Interestingly, an additional weak band is observed at 3650 cm^{-1} that we also assign to the stretch vibrations of OH groups of the metal-oxo clusters. The different frequency can be explained from the fact that the lattice contains defects caused by missing linkers, thus yielding a different coordination environment of the OH groups in the clusters."

Reviewer's question

There is no discussion of how the obtained results can be useful to researchers working with MOFs who are not specialists in spectroscopy.

Authors' response

We thank the reviewer for this comment. The preparation of UiO-66 membranes on solid substrates is a topic of strong current interest. Recently, preparation of UiO-66 membranes and its derivatives on gold substrates (J. Am. Chem. Soc. 2018, 140, 4812-4819), sapphire rods (*Eur. J. Inorg. Chem.* 14, 2094–2099 (2017), α-Al_2O_3 disks (J. Membr. Sci. 2022, 653, 120496) and ZrO_2@γ-Al_2O_3 fibers (Nat Commun 13, 266 (2022) have been reported. The present study presents a new protocol for preparation of polycrystalline films of UiO-66 on flat c-sapphire substrates with predominate [111] orientation. The advantage of the protocol presented in our study is that the samples are prepared in a single step solvothermal process which does not involve preparation of the precursors such as Zr-based clusters. Additionally, to the best of our knowledge, no protocols using c-sapphire substrates have been reported. The sapphire substrates have a broad transparency window in the optical frequency range which is promising for applications in photocatalysis and electrochemistry. Furthermore, the membranes prepared according to the protocol contain high concentration of missing linkers. Highly defective materials have been proven applicable in absorptive removal of pollutants (ACS Sustainable Chem. Eng. 2019, 7, 6619-6628) gas separation (J. Membr. Sci. 2022, 653, 120496) and catalysis (Applied Energy 277, 2020, 115560).

Another broadly useful result of our work is that we observe that the thermalization of the energy of the excited vibrations results in an immediate red-shift of the vibrational bands of the carboxylate group. Recently, for a large variety of carboxylate-based MOFs similar red-shifts have been shown to indicate a loosening of the metal carboxylate linkages (J. Am. Chem. Soc. 2020, 142, 19291 - 19299). Hence, our results show that any heat dissipated in the MOF will lead to a loosening of the MOF linkages on a picosecond timescale. This information is important for further mechanistic studies involving MOFs with a broad range of (photo)catalytic functionalities.

Actions taken

We added the following sentences to the description of our preparation method:

The preparation of UiO-66 membranes on solid substrates is a topic of strong current interest. Recently, the preparation of UiO-66 membranes and its derivatives on gold substrates (J. Am. Chem. Soc. 2018, 140, 4812-4819), sapphire rods (*Eur. J. Inorg. Chem.* 14, 2094–2099 (2017), α-Al_2O_3 disks (J. Membr. Sci. 2022, 653, 120496) and ZrO_2@γ-Al_2O_3 fibers (Nat Commun 13, 266 (2022) have been reported. However, to the best of our knowledge, no protocols using flat c-sapphire substrates have been reported.

We added the following paragraph to the discussion section:

"The observation of a hot state signal means that thermalization of the energy of the excited vibrations results in an immediate red-shift of the vibrational bands of the carboxylate group. Recently, for a large variety of carboxylate-based MOFs similar red-shifts have been shown to indicate a loosening of the metal carboxylate linkages (J. Am. Chem. Soc. 2020, 142, 19291 - 19299). Hence, our results indicate that any heat dissipated in the MOF will lead to a loosening of the MOF linkages on a picosecond timescale. This information is important for further mechanistic studies involving MOFs with a broad range of (photo)catalytic functionalities."

We added the following paragraph to the summary of the manuscript:

"In summary, in this work we presented a new protocol for preparation of polycrystalline films of UiO-66 on flat sapphire c-substrates. These metal organic frameworks consist of Zr^{4+} ions connected by terephthalate linkers. The advantage of this protocol is that the samples are prepared in a single step solvothermal process which does not involve preparation of the precursors such as Zr-based clusters. Additionally, to the best of our knowledge, no protocols using c-sapphire substrates have been reported. The sapphire substrates have a broad transparency window in the optical frequency range which is promising for applications in photocatalysis and electrochemistry. Furthermore, the membranes prepared according to the protocol contain high concentration of missing linkers. Highly defective materials have been proven applicable in absorptive removal of pollutants (ACS Sustainable Chem. Eng. 2019, 7, 6619-6628) gas separation (J. Membr. Sci. 2022, 653, 120496) and catalysis (Applied Energy 277, 2020, 115560)."

Reviewer's question

In the last paragraph of the paper, after discussing the effect of defects on the spectra, the authors suddenly start a text that looks like Conclusions.

Authors' response and actions taken

We thank the reviewer for pointing this out. We have created a clearer division of the main manuscript Discussion and Conclusions subsections.

Reviewer 2

Reviewer's question

1) To make the paper more accessible to non-experts, a diagram of the relevant molecular structures would be helpful in following the spectroscopic interpretations.

Authors' response and actions taken

Following the suggestion of the reviewer we added the molecular structures of the terephthalate linker and the $Zr_6O_4(OH)_4$ metal-oxo cluster to Figure 1.

Reviewer's question

2) The formatting of Figure 2 was not ideal in my version – in particular the left-hand side of the 2D-IR spectra appear to be cut off by the axis labels

Authors' response and actions taken

We thank the reviewer for pointing this out. We corrected the lay-out of Figure 2.

Reviewer's question

3) The assignment of the features left at long waiting times to a 'hot state' (assumed to mean generic thermal heating following vibrational relaxation) seems reasonable given the observed dynamics. It is however noticeable that the spectra at waiting times of 200 ps and 7 ps are virtually identical to the one recorded at 2 ps, except for some changes in amplitude caused by energy transfer. This suggests that the 'hot state' has an identical spectrum to that of the vibrationally excited material and makes it hard to differentiate the proposed mechanism of relaxation and thermal heating from a scenario in which vibrational relaxation proceeds via fast energy transfer (1.3 ps) and a very slow (> 200ps) vibrational relaxation. How do the authors rule out the second scenario? A study of the FTIR spectrum of the membrane as a function of temperature could be used to show the result of increased T on the carboxylate bands for comparison with the 200 ps data. Ordinarily it would be expected that a carboxylate band would shift to higher frequencies at elevated temperature, which would give a different spectral profile to that of a vibrationally-excited one.

Authors' response

We thank the reviewer for the interesting question. The alternative relaxation mechanism proposed by the reviewer (fast energy transfer with a time constant of 1.3 ps followed by very slow vibrational relaxation with time constant > 200 ps) would lead to a growth of the υ_s -> υ_{as} cross-peak signal ($\omega_{excitation}$ = 1395 cm^{-1} , $\omega_{detection}$ = 1585 cm^{-1}). However, as seen in Figure 3b, we do not observe a growing component in the cross-peak, and only a decaying component is observed. Thus, energy transfer with a time constant of 1.3 ps can be ruled out.

To further corroborate the thermal nature of the transient signals at long waiting time, we have performed a study of the temperature dependence of the membrane FTIR spectrum, as suggested by the reviewer, and we compared the results to the transient absorption spectra at waiting times >10 ps. In Figure 10 of the Supporting Information we show linear absorption spectra detected at elevated temperatures in the frequency region of the ν_{as} vibration. As can be seen from Figure 10 of the Supporting Information, increasing the temperature leads to a shift of the maximum of the

band to lower frequencies without significantly changing the absorption cross-section. The magnitude of the shift is on the order of a few cm^{-1} and proportional to the temperature increase. By subtracting the spectrum detected at room temperature from the spectra detected at elevated temperature, we obtain the thermal difference spectra that we show in Figure 11 of the Supporting. Comparison of Figure 11 of the Supporting Information with Figure 8 of the Supporting Information shows that the shape of the thermal difference spectrum is very similar to the difference absorption spectra observed at waiting times >10 ps with 2D-IR spectroscopy. As the shift of the band is much smaller than the bandwidth, subtraction of the spectra yields an anti-symmetric dispersive shape of which the amplitude scales with the magnitude of the shift. This shape is similar to that of the excited state spectrum because the diagonal anharmonicity is also much smaller than the width of the absorption band.

Actions taken

See actions taken for the following question

Reviewer's question

4) Related to 3) it is stated in the discussion that 'the final signal (the hot-state end level) is determined by the total energy absorbed from the excitation pulse.' Has this been experimentally verified?

Authors' response

We thank the reviewer for the interesting question. Following the suggestion of the reviewer, we calculated the temperature change in the excited volume resulting from relaxation and thermal equilibration of the femtosecond vibrational excitation. To this purpose, we used the total pump pulse energy of ~5 μJ and the spectrum that we show in Figure 13 of the Supporting Information, a beam diameter at the sample position of 200 μm, a sample absorbance detected from FTIR spectra and sample thickness of ~700 nm as determined from profilometry measurements (see Figure 2 of the Supporting Information), the UiO-66 heat capacity of 1000 J/(K*kg) (*ACS Appl. Mater. Interfaces* 2019, 11, 42, 38697–38707), and finally a density of 1200 kg/m^3 (*Nat Commun* 10, 2345 (2019). With these parameters, the increase of the temperature is calculated to be ~10K for excitation of the υ_a vibration, and ~20K in the case of excitation of the υ_s vibration.

In Figure 12 of the Supporting Information, we show pump-averaged transient spectra corresponding to the diagonal υ_{as} peak ($\omega_{excitation} = \omega_{detection} = 1585$ cm^{-1}) and the υ_s -> υ_{as} cross-peak ($\omega_{excitation} = 1395$ cm^{-1} , $\omega_{detection} = 1585$ cm^{-1}). Comparing Figures S11 and S12 it is clearly seen that the transient absorption spectra observed at long waiting times shown in Figure 12 of the Supporting Information match quite well with thermal difference spectra in Figure 11 of the Supporting Information. This corroborates the assignment of the transient absorption spectra at waiting times >10 ps to a heating effect, i.e., completed vibrational relaxation and thermal equilibration in the UiO-66 film.

Actions taken

We added a new section to the Supporting Information "Analysis of the temperature dependence of the FTIR spectra of the UiO-66 membrane and interpretation of the signals at long waiting times". To this section we added Figure 10 with the caption "Infrared absorption spectra of UiO-66 membranes at elevated temperatures", Figure 11 with the caption "Thermal difference of the infrared absorption spectra of UiO-66 membranes displayed in Figure 10", Figure 12 with the caption "Isotropic transient absorption spectra measured for UiO-66 membranes at waiting time T = 200 ps for υ_s -> υ_{as} cross-peak signal ($\omega_{excitation}$ = 1395 cm^{-1}, $\omega_{detection}$ = 1585 cm^{-1}) (blue) and the diagonal υ_{as} signal ($\omega_{excitation}$ = $\omega_{detection}$ = 1585 cm^{-1}) (orange)", Figure 13 with the caption "Spectral profile of the excitation pulse".

We also added the following text:

"To corroborate the thermal nature of the transient signals at long waiting time, we have performed a study of temperature dependence of the membrane FTIR spectrum. In Figure 10 we show the spectra detected at elevated temperatures in the frequency region corresponding to the υ_{as} vibration. As can be clearly seen from Figure 10, increasing the temperature leads to a shift of the maximum of the band to lower frequencies without significantly changing the absorption cross-section. The magnitude of the shift is on the order of a few cm^{-1} and proportional to the temperature increase. By subtracting the spectrum detected at room temperature from the spectra detected at elevated temperature, we obtain the thermal difference spectra that we show in Figure 11. Comparison of Figure 11 with Figure 8 shows that the shape of the thermal difference spectrum is very similar to the difference absorption spectra observed at waiting times >10 ps with 2D-IR spectroscopy. As the shift of the band is much smaller than the bandwidth, subtraction of the spectra yields an anti-symmetric dispersive shape of which the amplitude scales with the magnitude of the shift. This shape is similar to that of the excited state spectrum because the diagonal anharmonicity is also much smaller than the width of the absorption band.

We calculated the temperature change in the excited volume using the following expression: $\Delta T = E_{absorbed}/(c*m)$, where ΔT is the temperature change, $E_{absorbed}$ - excitation energy absorbed by the sample, c is the heat capacity and m is the mass of the volume that is excited. The absorbed energy was calculated by integration:

$$\int_{\omega_{min}}^{\omega_{max}} d\omega(1-10^{-A(\omega)})S(\omega)$$

Where $A(\omega)$ is the sample absorbance determined from FTIR spectra, $S(\omega)$ is the spectrum of the excitation pulse shown in Figure 13, ω_{min} = 1350 cm^{-1} , ω_{max} = 1450 cm^{-1} and ω_{min} = 1540 cm^{-1} , ω_{max} = 1610 cm^{-1} were used for υ_s and υ_{as} vibrations respectively. The excitation pulse energy of ~5 μJ was used for normalization of the integral. To calculate the mass of the excited volume we used the beam diameter at the sample position of ~200 μm, and the sample thickness of 700 nm (see Figure 2), UiO-66 density of 1200 kg/m^3 (*Nat Commun* 10, 2345 (2019) and a heat capacity of 1000 J/(kg*K) (*ACS Appl. Mater. Interfaces* 2019, 11, 42, 38697–38707). With these parameters, we calculate the increase in temperature ~10K for excitation of the υ_{as} vibration and ~20K in the case of excitation of the υ_s vibration.

In Figure 12, we show pump-averaged transient spectra corresponding to the diagonal υ_{as} peak ($\omega_{excitation} = \omega_{detection} = 1585$ cm^{-1}) and the υ_s -> υ_{as} cross-peak ($\omega_{excitation} = 1395$ cm^{-1} , $\omega_{detection} = 1585$ cm^{-1}). Comparing Figures S11 and S12 it is clearly seen that the transient absorption spectra observed at long waiting times shown in Figure 12 match quite well with thermal difference spectra in Figure 11. This result corroborates the assignment of the transient absorption spectra at waiting times >10 ps to a heating effect, i.e. completed vibrational relaxation and thermal equilibration in the UiO-66 film."

Reviewer's question

There are several very long passages of text (e.g. p4&5), which would be made easier to read by greater use of paragraphs.

Authors' response and actions taken

We thank the reviewer for the suggestion. We have used more paragraphs in the Discussion section of the main manuscript text.
